# Differential gene expression and immune cell infiltration in maedi-visna virus-infected lung tissues

**DOI:** 10.1186/s12864-024-10448-2

**Published:** 2024-05-30

**Authors:** Xiaona Shi, Yufei Zhang, Sixu Chen, Xiaoyue Du, Pei Zhang, Xujie Duan, Hui Fang, Shuying Liu

**Affiliations:** 1 College of Veterinary Medicine, Inner Mongolia Agricultural University, Zhao Wu Da Road No. 306, Hohhot, 010018 China; 2 Inner Mongolia Key Laboratory of Basic Veterinary Science, Hohhot, 010018 China; 3 Key Laboratory of Clinical Diagnosis and Treatment Technology in Animal Disease, Ministry of Agriculture, Hohhot, 010018 China

**Keywords:** Maedi-Visna virus, Ovine progressive pneumonia, Sheep, RNA-seq, Differential expression, Pathogenesis, Immunopathogenesis

## Abstract

**Background:**

Maedi-visna virus (MVV) is a lentivirus that infects monocyte/macrophage lineage cells in sheep, goats, and wild ruminants and causes pneumonia, mastitis, arthritis, and encephalitis. The immune response to MVV infection is complex, and a complete understanding of its infection and pathogenesis is lacking. This study investigated the in vivo transcriptomic patterns of lung tissues in sheep exposed to MVV using the RNA sequencing technology.

**Result:**

The results indicated that 2,739 genes were significantly differentially expressed, with 1,643 downregulated genes and 1,096 upregulated genes. Many variables that could be unique to MVV infections were discovered. Gene Ontology analysis revealed that a significant proportion of genes was enriched in terms directly related to the immune system and biological responses to viral infections. Kyoto Encyclopedia of Genes and Genomes analysis revealed that the most enriched pathways were related to virus-host cell interactions and inflammatory responses. Numerous immune-related genes, including those encoding several cytokines and interferon regulatory factors, were identified in the protein-protein interaction network of differentially expressed genes (DEGs). The expression of DEGs was evaluated using real-time polymerase chain reaction and western blot analysis. *CXCL13*, *CXCL6*, *CXCL11*, *CCR1*, *CXCL8*, *CXCL9*,* CXCL10*, *TNFSF8*, *TNFRSF8*, *IL7R*, *IFN-γ*, *CCL2*, and *MMP9* were upregulated. Immunohistochemical analysis was performed to identify the types of immune cells that infiltrated MVV-infected tissues. B cells, CD4^+^ and CD8^+^ T cells, and macrophages were the most prevalent immune cells correlated with MVV infection in the lungs.

**Conclusion:**

Overall, the findings of this study provide a comprehensive understanding of the in vivo host response to MVV infection and offer new perspectives on the gene regulatory networks that underlie pathogenesis in natural hosts.

**Supplementary Information:**

The online version contains supplementary material available at 10.1186/s12864-024-10448-2.

## Background

A genetically diverse group of lentiviruses, known as small ruminant lentiviruses (SRLVs), belongs to the family Retroviridae and includes ovine maedi-visna virus (MVV) and caprine arthritis encephalitis virus (CAEV) [1]. Maedi-visna (MV), also referred to as ovine progressive pneumonia (OPP), is an incurable viral disease in sheep [2–4] characterized by a prolonged incubation period and permanent infection. Although the clinical condition of OPP has substantial effects on animal welfare and production, symptoms typically do not appear for years after the infection is established owing to the lengthy latent phase of SRLVs. Moreover, animals cannot be immunized against the disease, and there is no cure for this lifelong chronic disease.

MV is a pandemic disease common in countries with considerable sheep farming [4–11]. MV disease was discovered in China in 1984 [12–14]. It was initially believed to be a contagious disease that presented herd and individual seroprevalence rates of 100% and 4.6–50.0%, respectively [15, 16]. The disease is classified as a notifiable terrestrial animal disease by the World Organization for Animal Health; it has also been categorized as a Class III animal disease by the Ministry of Agriculture and Rural Affairs of the People’s Republic of China. Although MV disease is difficult to identify considering clinical signs, molecular methods and specific antibodies against MVV in blood samples are the most effective approaches for diagnosing MV in herds.

MVV mainly infects blood monocytes/macrophages and dendritic cells, although it may be present in various other tissues. The pulmonary lesions observed in sheep infected with MVV include lymphoid interstitial pneumonia and lymphocytic alveolitis [17–19]. The lungs of MVV-infected sheep with lymphoid interstitial pneumonia are twice to thrice heavier than healthy lungs and exhibit a consistently firm texture and a white or gray color [20–23]. Thickening of the interalveolar septa due to the infiltration of mononuclear cells, smooth muscle hyperplasia, and fibrosis has been observed under a microscope [23–25]. Additionally, numerous lymph follicles with active germinal centers occur near the bronchioles and small vessels [26, 27]. In severe cases, the disease results in almost obliterated alveoli as macrophages infiltrate the lumen. However, the molecular mechanisms underlying MVV-associated lymphoid interstitial pneumonia are not understood.

Animal lentivirus infections have been used as models for human immunodeficiency virus (HIV) infection ever since human lentiviruses were discovered to be the primary cause of acquired immunodeficiency syndrome in humans [28–30]. Consequently, enormous progress has been made in understanding the biology and pathology of animal lentiviruses. Therefore, MV has been the most studied sheep disease over the past three decades [2, 4, 14, 25, 31, 32]. Previous MVV studies have focused on fundamental cellular and molecular issues. However, there is a need to understand the intricate cellular and molecular interactions leading to MVV infections and symptom development.

This study used RNA sequencing (RNA-seq) to identify the factors associated with MVV infection in the host. Differentially expressed cellular mRNAs were identified, and details on the associated genes, mechanisms, and pathways were deduced through bioinformatics studies and confirmed through experiments. The results of this study will enhance our understanding of the interactions between the virus and its host and the molecular processes underlying MV development.

## Materials and methods

### Animals

This study used 20 adult Dorpe sheep aged from 2 to 4 years at various stages of natural MVV infection. The samples of sheep used in this study were collected from a sheep breeding farm in Inner Mongolia, China. Using an Enzyme-Linked ImmunoSorbent Assay (ELISA) (ID Screen MVV/CAEV Indirect-Screening test, ID.Vet Innovative Diagnostics, Montpellier, France), the animals were classified into two groups considering their MVV infection status (seronegative or seropositive), and their clinical outcome (asymptomatic and diseased) was also recorded (Table 1). For the RNA-seq analysis, six animals were selected, consisting of three sheep without antibodies (seronegative group) and three sheep with lung lesions and positive antibodies (lesion group). Additionally, we excluded cases of mixed infections caused by multiple pathogenic microorganisms, including *Pasteurellosis, Streptococcus pneumoniae*, *Mannheimia haemolytica*, *Mycoplasmosis*, morbillivirus), parainfluenza viruses, and jaagsiekte sheep retrovirus), using polymerase chain reaction (PCR) or quantitative reverse transcriptome polymerase chain reaction (RT-qPCR) techniques. The animals were euthanized via an overdose of barbituric acid (1,306, Damao Chemical Reagent Factory, Tianjin, China) administered intravenously, followed by exsanguination. Approval and licensure for the entire experimental protocol were granted by the Specialized Committee on Scientific Research and Academic Ethics of Inner Mongolia Agricultural University, China (approval number: 2020–007) [33].

**Table 1:** Results of ELISA detection of sheep serum

	Ear tag number	OD_450nm_	State
1	04399	0.880	+
2	1715	0.868	+
3	80,375	0.910	+
4	01079	0.802	+
5	00473	0.669	+
6	8847	0.574	+
7	12,433	0.261	+
8	10,123	0.231	+/-
9	08009	0.091	-
10	08653	0.113	-
11	05695	0.169	-
12	01650	0.058	-
13	07177	0.201	-
14	10,206	0.072	-
15	00999	0.065	-
16	01570	0.095	-
17	03453	0.136	-
18	11,295	0.078	-
19	12,535	0.157	-
20	00511	0.211	-

*Note* Average OD450nm value of positive control = 0.49; Average OD450nm value of negative control = 0.058

### Histopathological investigation

Tissue samples collected from the infected lungs and control groups were preserved in a solution of 10% neutral buffered formalin (Thermo Fisher Scientific, Waltham, MA, USA) and prepared using standard pathological protocols. Next, 4 μm-thick sections were subjected to hematoxylin and eosin staining (Thermo Fisher Scientific) and examined for MV lesions [33].

Lung specimens were further assessed using an immunofluorescence assay to detect the SRLV p28 capsid antigen [34]. After removing the wax with xylene and rehydrating the sections with alcohol, the slides were microwaved in ethylenediaminetetraacetic acid buffer (pH 6.0 [Thermo Fisher Scientific]) at 600 W for 9 min and at 400 W for 7 min. The sections were then rinsed thrice with phosphate-buffered saline (PBS [pH 7.4 {Thermo Fisher Scientific}]) and sealed with 3% bovine serum albumin (GC305010, Servicebio, Wuhan, China) for 30 min. Next, the sections were placed in a humid chamber, exposed to primary antibodies (MAb CAEV-63, CAEV-Co, MVV, OPPV IgG1, CAEP5A1, and VMRD [Pullman, WA, USA]) that had been diluted to 1:100, and incubated overnight at 4 ℃ [35]. After washing with PBS, the sections were incubated with a 1:400 dilution of Alexa Fluor 488-conjugated Goat Anti-Mouse IgG (H + L) (GB25301, Servicebio) and then subjected to 4′,6-diamidino-2-phenylindole staining (G1012, Servicebio) to stain the nuclei; finally, the sections were observed under a fluorescence microscope (Biobase group, Shandong, China).

### MV provirus detection

Whole blood and lung samples were used for genomic DNA extraction using the TIANamp Genomic DNA Kit (DP304, TIANGEN, Beijing, China). DNA purity was verified using an enzyme labeling instrument and agar gel electrophoresis, resulting in an A 260:280 ratio of approximately 1.8-2.0 for almost all the samples. Water without DNAase or RNAase was used as the negative control. The MV provirus was detected via PCR of the long terminal repeat (LTR) region. To amplify a 291 bp DNA fragment in the LTR region, the following primers were used: forward 5´-TGA CAC AGC AAA TGT AAC CGC AAG-3´ and reverse 5´-CCA CGT TGG GCG CCA GCT GCG AGA-3´ [36]. The final volume of the reaction mix was 25 µL and comprised 20 ng genomic DNA, 12.5 µL Green Taq Mix (P131-01, Vazyme Biotech Co., Ltd., Nanjing, China), 1 µl forward primer, and 1 µL reverse primer. The thermal cycling conditions were as follows: one cycle of initial denaturation at 95 ℃ for 5 min, followed by 30 cycles of denaturation at 95 ℃ for 30 s, annealing at 63 ℃ for 30 s, extension at 72 ℃ for 30 s, and final extension at 72 ℃ for 5 min. The amplified PCR products were examined via electrophoresis on 2% agarose gels (Agarose Bead Technologies, Madrid, Spain) and observed under ultraviolet light. A PCR product derived from an MVV-positive sample was generated to create a positive control for PCR amplification; the negative control comprised DNA from an MVV-seronegative sample.

### PCR amplification, sequencing and sequence analysis

The primers of gag and env regions were designed based on NM1111 (MW248464) using Primer Premier software version 5. To amplify a 1399 bp DNA fragment in the *gag* region, the following primers were used: forward 5´-GAA GCA AGG CTC GAG AGA GAA-3´ and reverse 5´-TTA TAA CAT AGG GGG TGC GGA-3´. The *env* primers were used: forward 5´-ATGGCAGAAAGGGAAAAACAGAAA-3´ and reverse 5´-TGCGTACTCCGTCTCTACTT-3´, which amplified a 2982 bp fragment. The target fragments were amplified by PCR and visualized on 1% agarose gel, and then purified using a EasyPure Quick Gel Extraction Kit (EG101-01, Trans, Beijing, China). The purified product was ligated with pEASY-Blunt Zero Cloning Vector (CB501-01, Trans, Beijing, China) and sequenced.

Phylogenetic analyses of sequencing data from the gag and env regions with the whole-genome sequences of a total of 78 SRLVs belonging to genotypes A-E in GenBank (http://www.ncbi.nlm.nih.gov/genbank/) using MEGA v7.0.21 software by Claustral W algorithm, respectively. The phylogenetic tree was inferred using the neighbor-joining method. Bootstrap values were estimated for 1,000 replicates and the evolutionary distances were computed using the p-distance method.

### Transmission electron microscopy

Lung samples from sheep that tested positive for MVV using ELISA and PCR/sequencing were prepared for transmission electron microscopy (TEM). The specimens were divided into 1 mm^3^ blocks and then immersed in a chilled solution containing 2.5% glutaraldehyde diluted in a 0.1 M phosphate buffer (PB [pH = 7.4; Thermo Fisher Scientific]) for 5 h. Then, the specimens were washed thrice in 0.1 M PB, treated with 1% osmium tetroxide for 2 h in the absence of light, and dehydrated with a sequence of ethanol concentrations and twice with acetone. Subsequently, the specimens were embedded in a 2:1 and 1:2 mixture of acetone and Epon 812, respectively, for 4 h and then immersed in fresh 100% Epon 812 twice for 12 h. The last step involved the polymerization of the blocks in fresh Epon resin for 48 h in an oven at 65 ℃. Next, 50–70 nm-thick sections were obtained using an ultramicrotome (EM UC7; Leica, Wetzlar, Germany) and subsequently placed onto 200-mesh copper grids equipped with carbon-stabilized formvar support films (Labtech International, Batam, Indonesia). The sections were immersed in 2% uranium acetate for 15 min and lead citrate for 5 min, washed with distilled water, air-dried, and examined under an electron microscope (H-7650; HITACHI, Tokyo, Japan).

### Tissue collection, RNA extraction, and RNA sequencing

Sterile collection of lung tissue was performed for all animals, and the samples were preserved in RNA-later solution (AM7020, Invitrogen, Carlsbad, CA, USA) at -80 ℃ until analysis. Total RNA was extracted from lung tissue using TRIzol (15,596,026, Invitrogen, Carlsbad, CA, USA). After adding chloroform, RNA was separated from the top aqueous layer using isopropanol, rinsed with ethanol, suspended in RNase-free water, and finally stored at -80 ℃. RNA purity and concentration were assessed using a NanoPhotometer spectrophotometer (Thermo Fisher Scientific) and a Qubit 2.0 Fluorometer (Invitrogen), respectively. RNA integrity was evaluated using an Agilent 2100 Bioanalyzer (Agilent Technologies, Palo Alto, CA, USA) and RNase-free agarose-gel electrophoresis. After isolating the total RNA, mRNA was enriched using oligo dT beads (Thermo Fisher Scientific), fragmented into smaller pieces using a fragmentation buffer (Thermo Fisher Scientific), and converted to cDNA through reverse transcription using random primers. Subsequently, second-strand cDNA was synthesized, and fragments were purified using the QiaQuick PCR extraction kit (Qiagen, Venlo, Netherlands). The fragments were then subjected to end repair, where an A base was added, followed by ligation to Illumina sequencing adapters. Agarose-gel electrophoresis was used to select the sizes of the ligation products, which were then PCR-amplified and sequenced on Illumina Novaseq6000 at Gene Denovo Biotechnology Co., Guangzhou, China.

### Transcriptome mapping and analysis of differentially expressed genes

To obtain clean reads of superior quality, the reads were subjected to additional filtration using fastp version 0.18.0 (Github, San Francisco, CA, USA) [37]; the reads that were mapped to rRNA were subsequently eliminated using the short reads alignment tool Bowtie2 (Github) [38]. The remaining filtered reads were used to determine gene abundance in the assembly process. HISAT2.2.4 software (Github) [39] was used to map the processed reads to the Oar_v4.0 (ncbi_GCF_000298735.2 [Github]) reference genome by employing the default parameters. StringTie v1.3.1 (Github) [33, 35] was used to assemble the mapped reads of each sample in a reference-based manner. The RNA-Seq by Expectation-Maximization software [39] was used to calculate the fragment per kilobase of transcript per million mapped reads (FPKM) value to quantify the expression levels and variations in each transcription region. Genes/transcripts meeting the criteria of a false discovery rate (FDR) below 0.05 (edgeR) and a fold change exceeding 2 were identified as differentially expressed and subsequently subjected to additional annotation analysis.

### Gene ontology enrichment and pathway analysis

The compiled transcripts were annotated using BLASTx (National Center for Biotechnology Information (NCBI), Bethesda, MD, USA) against protein databases, such as the NCBI, non-redundant, Swiss-Prot, Kyoto Encyclopedia of Genes and Genomes (KEGG), Clusters of Orthologous Groups, and gene ontology (GO), with an e-value threshold of 1e-5. Sets of differentially expressed genes (DEGs) were subjected to GO and pathway analyses. Next, the gene lists exhibiting differential expression were used to map the genes associated with each GO category, and their ranking was established using the F statistic [40]. Cluster 3.0 (Stanford University, Stanford, CA, USA) with the settings “-g 7 -e 7 -m a” was used for the clustering analysis [33]. Pathway functional analysis was performed using David software v6.8 (Fisher’s exact test) and KEGG, along with the KEGG mapper search pathway tools [41]. Significantly enriched pathways were identified by analyzing the p-values obtained using Fisher’s exact test.

### Quantitative reverse transcription-polymerase chain reaction analysis

A Total RNA Miniprep Kit (AP-MN-MS-RNA-50, Axygen, Silicon Valley, CA, USA) was used to isolate total RNA from frozen lung samples for quantitative analysis. The isolation was performed according to the manufacturer’s instructions. Afterward, 1 µg of RNA was reverse transcribed to first-strand cDNA using random primers and the HiScript III 1st Strand cDNA Synthesis Kit (+ gDNA wiper [R312-01 {Vazyme Biotech Co., Ltd., Nanjing, China}]). ChamQ Universal SYBR qPCR Master Mix (Q711-02, Vazyme Biotech Co., Ltd., Nanjing, China) was used for real-time PCR (qPCR) on a Real-Time PCR device (ABI7500, Applied Biosystems, Waltham, MA, USA) using the primer sequences listed in Supplementary Table 1. The *β*-actin gene served as the housekeeping gene. The cycling conditions were as follows: initial denaturation at 95 ℃ for 10 min, followed by 40 cycles of amplification at 95 ℃ for 10 s and 60 ℃ for 45 s. PCR melting curves were carefully examined to verify the presence of a single PCR amplicon for each amplification, and the product was verified via nucleotide sequencing. The assay efficiency was evaluated by analyzing the amplification efficiencies through calibration curves and verifying that the slope of the logarithm of the input versus the △Cq plot was less than 0.1. A minimum of three distinct frozen lung samples were examined from each group, and each specimen was analyzed in triplicate. The 2^−ΔΔCt^ method was utilized to determine quantitative variations and relative fold changes.

### Western blot analysis

Protein samples were prepared using radioimmunoprecipitation assay lysis buffer (Thermo Fisher Scientific) (containing 50 mM Tris [pH 7.4], 150 mM NaCl, 1% NP-40, and 0.5% sodium deoxycholate), along with a combination of protease inhibitors, phosphatase inhibitors, and phenylmethylsulfonyl fluoride from the Tissue or Cell Total Protein Extraction Kit (C510003, Sangon Biotech Co., Ltd., Shanghai, China). Protein concentrations were assessed by spinning the extracts at a speed of 14,000 rpm for 15 min at 4 ℃. Quantification was performed using a Bicinchoninic Acid Protein Assay Kit (C503021; Sangon Biotech Co., Ltd.). Total protein (50 µg) was separated through sodium dodecyl-sulfate polyacrylamide gel electrophoresis and detected using traditional protocols and the following antibodies: CXCL8/IL-8 polyclonal antibody (27095-1-AP, Proteintech, Wuhan, China), interferon-gamma (IFN-γ) antibody (AF5183, Affinity Biosciences, Jiangsu, China), IL10 antibody (DF6894, Affinity Biosciences), MMP9 antibody (AF5228, Affinity Biosciences), CCL2/MCP-1 rabbit pAb (A7277, ABclonal Technology Co., Ltd., Wuhan, China), beta-actin monoclonal antibody (66009-1-Ig, Proteintech), HRP-conjugated Affinipure Goat Anti-Mouse IgG(H + L) (SA00001-1, Proteintech), and HRP-conjugated Affinipure Goat Anti-Rabbit IgG(H + L) (SA00001-2, Proteintech). Signals were measured using the ImageJ software (http://imagej.nih.gov/ij) National Institutes of Health and the Laboratory for Optical and Computational Instrumentation, University of Wisconsin, Madison, WI, USA).

### Immunohistochemistry

Paraffin Sect. (4 μm) were baked and then routinely dewaxed, 3% H_2_O_2_ was added, and then they were allowed to react. The tissues were incubated with a 1:200 dilution of CD4 polyclonal antibody (19068-1-AP, Proteintech, Wuhan, China), rabbit polyclonal CD8 alpha (ab4055, Abcam, Cambridge, USA), CD19 rabbit mAb (A19013, ABclonal Technology Co., Ltd.), and rabbit polyclonal antibody against CD68 (DF7518, Affinity Biosciences) overnight at 4 ℃. Subsequently, the sections were washed and incubated with a secondary antibody (1:200) for 1 h at room temperature. Immunoreactivity was visualized using diaminobenzidine (AR1022; Boster, Wuhan, China), and nuclear counterstaining was performed using hematoxylin. After removing moisture and achieving transparency, the slices were hermetically sealed for observation.

### Statistical analysis

The data were subjected to a one-way analysis of variance using the PRISM software (Prism software, Irvine, CA, USA), followed by Tukey’s multiple comparison test. The average ± standard error of the mean (SEM) was computed for each treatment group in separate experiments. A two-tailed Student’s *t*-test was employed to determine significant differences between the treatment and control groups (***p < 0.001, ns = non-significant at the 95% confidence level).

## Results

### Confirmation of MVV infection in pulmonary tissues

**Fig. 1:**
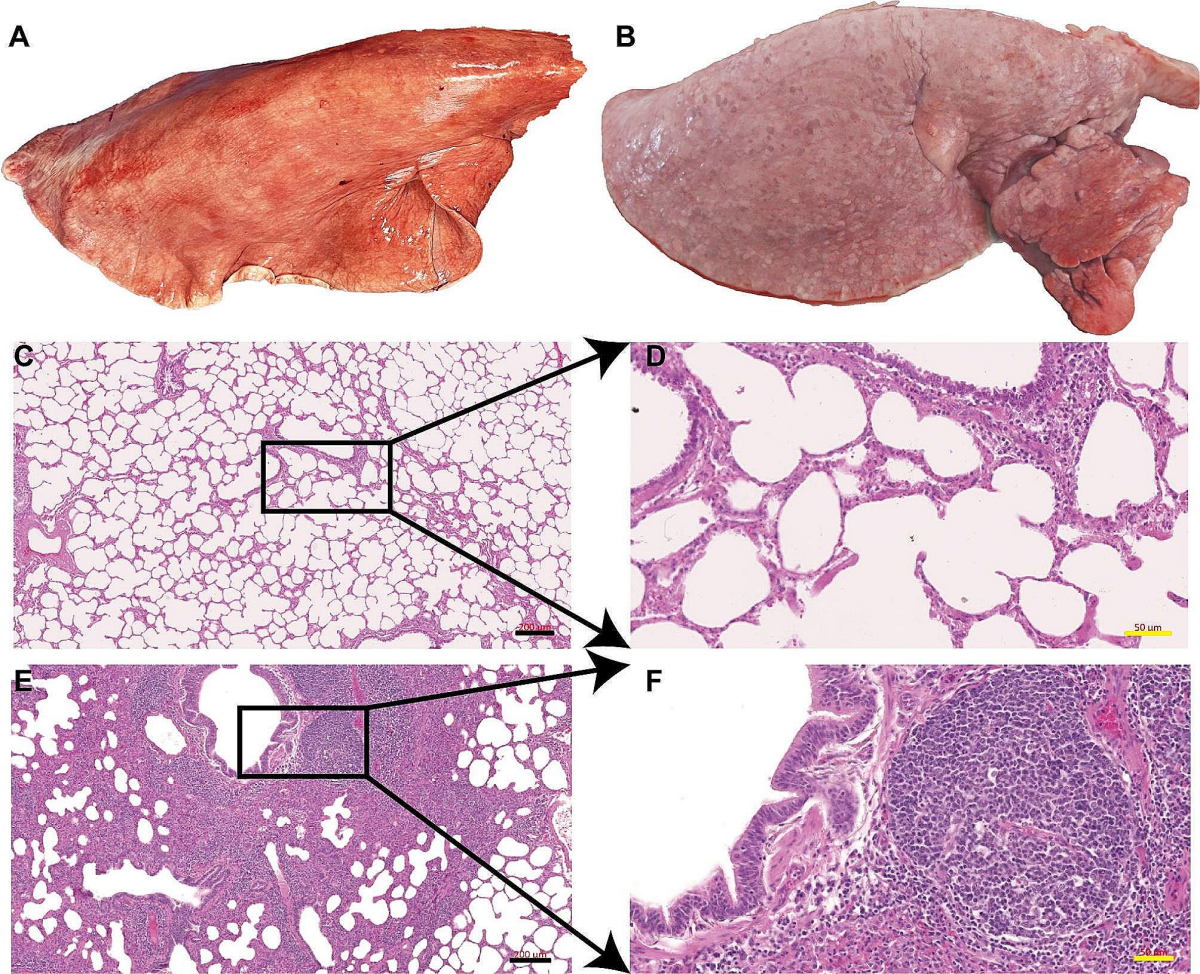
Histopathologic characteristics of Maedi-visna virus (MVV)-infected lungs. (**A**) Overall appearance of the lung from a healthy sheep; (**B**) appearance of Maedi-visna disease in an infected sheep lung sample; (**C** and **D**) microscopic examination of the lung from a healthy sheep (black bar, 200 μm; yellow bar, 50 μm); (**E** and **F**) microscopic view of MVV-infected lungs (black bar, 200 μm; yellow bar, 50 μm)

Necropsy findings in all three sheep revealed gross pulmonary lesions indicative of MVV infection, accompanied by secondary bronchopneumonia. The affected lungs exhibited a notable increase in weight relative to that in the normal lungs, with localized or multiple enlarged nodules and a distended appearance (Fig. 1A and B). Lung color varied from pink to white, and the affected lung tissues exhibited a firm texture and a dense consistency. Histopathological analysis of the lung tissue corroborated the macroscopic observations. The lung sections, shown in Fig. 1C, D, E, and F, consistently exhibited chronic, widespread, and intense lymphohistiocytic interstitial pneumonia. This condition was identified by the presence of perivascular and peribronchial lymphofollicular proliferations that often contained germinal centers. Lesions indicating MVV infection coincided with a substantial influx of neutrophils into the bronchioles and alveoli, suggesting the occurrence of secondary, multifocal, acute bronchopneumonia. The pulmonary parenchyma contained scattered lymphocytic proliferative nodules, mainly encircling the bronchi, bronchioles, and blood vessels. The abnormalities included lymphoid cells encircling the bronchioles and blood vessels and an increase in smooth muscles in the alveolar wall and terminal bronchioles.

### Detection of MVV in the lung sections

**Fig. 2:**
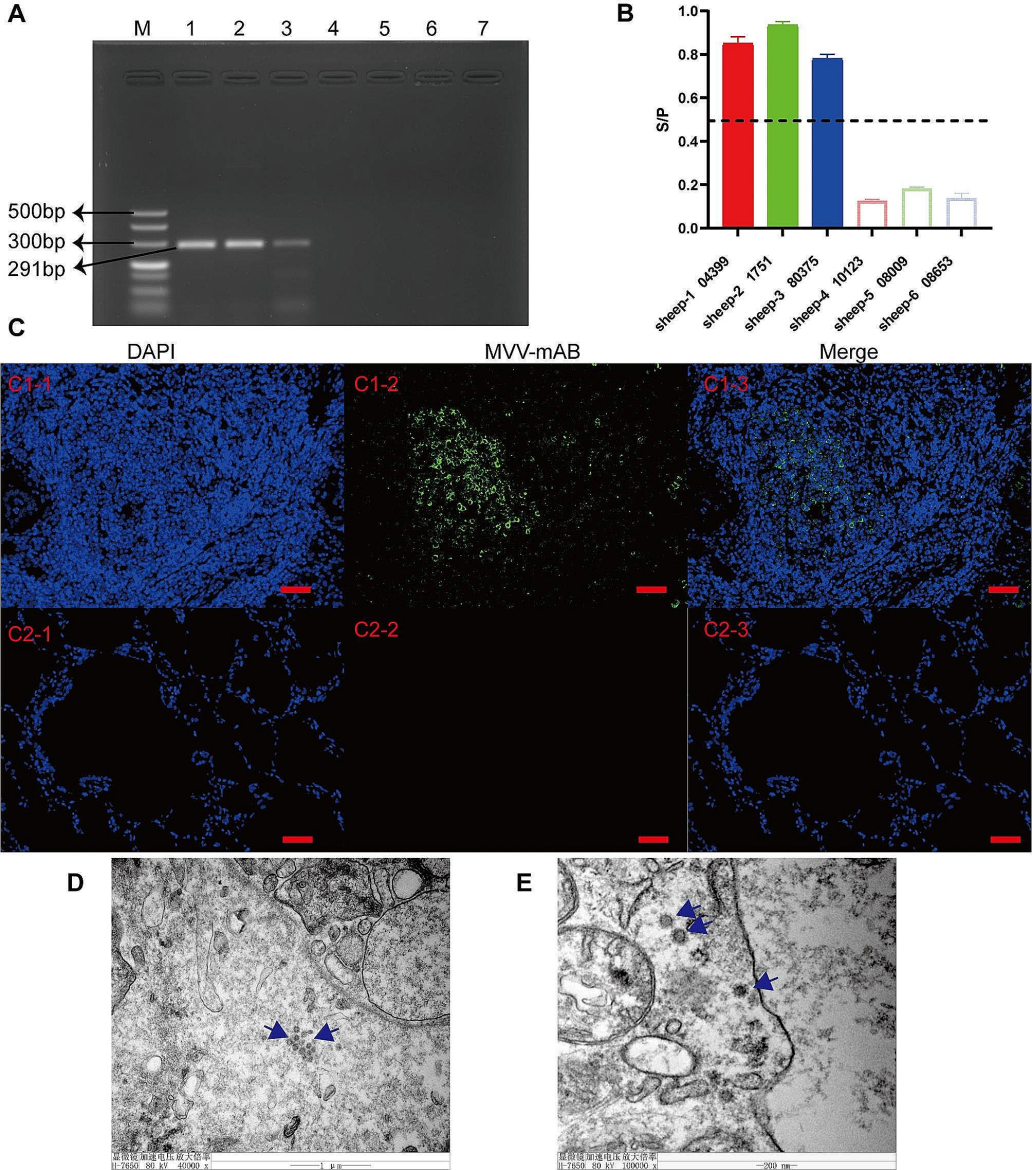
Identification of Maedi-visna virus (MVV) within the lungs of naturally infected sheep. (**A**) Polymerase chain reaction products were subjected to agarose-gel electrophoresis. Lane M presents 500 bp molecular weight markers; Lanes 1–3 present lung tissue samples from three MVV-infected sheep; Lanes 4–6 present lung tissue samples from three healthy sheep; and Lane 7 presents the negative control. (**B**) Enzyme-Linked ImmunoSorbent Assay for MVV antibody production. (**C**) Immunofluorescence of the capsid protein in the lungs of MVV-infected or -uninfected sheep (scale bar, 50 μm). (**D** and **E**) Transmission electron micrographs of MVV-infected lungs. (**D**) Viral buds can be observed on the outer membrane of macrophages infected with MVV (scale bar, 1,000 nm), whereas (**E**) intracytoplasmic particles are visible within the macrophages (scale bar, 200 nm)

PCR was used to detect proviral DNA sequences in the tissues to further diagnose MVV infection. PCR analysis of the three samples yielded amplicons of 291 bp in the LTR-DNA region of the MVV (Fig. 2A). Microscopic evaluation revealed MV lesions in the same cases. PCR analysis of the lungs of the three control animals revealed MVV-negative results. In this study, three PCR-positive sheep tested positive for MVV/CAEV with indirect ELISA (Fig. 2B). Serial sections of lung tissue were subjected to IF using mAb MVV. In all MVV-infected sheep, viral proteins were found in the cytoplasm of macrophages in the lungs (Fig. 2C). Sections from non-infected animals and negative control sections had no cytoplasmic labeling (Data not presented). TEM-based confirmation of seropositive and PCR/sequencing results corroborated ultrastructural changes in the MVV from lung samples and revealed the characteristic attributes of MVV and viral particles generated by macrophages (Fig. 2D). Moreover, the cytoplasm of MVV-infected cells contained virions (Fig. 2E).

### Phylogenetic trees inferred from *gag* and *env* regions

**Fig. 3:**
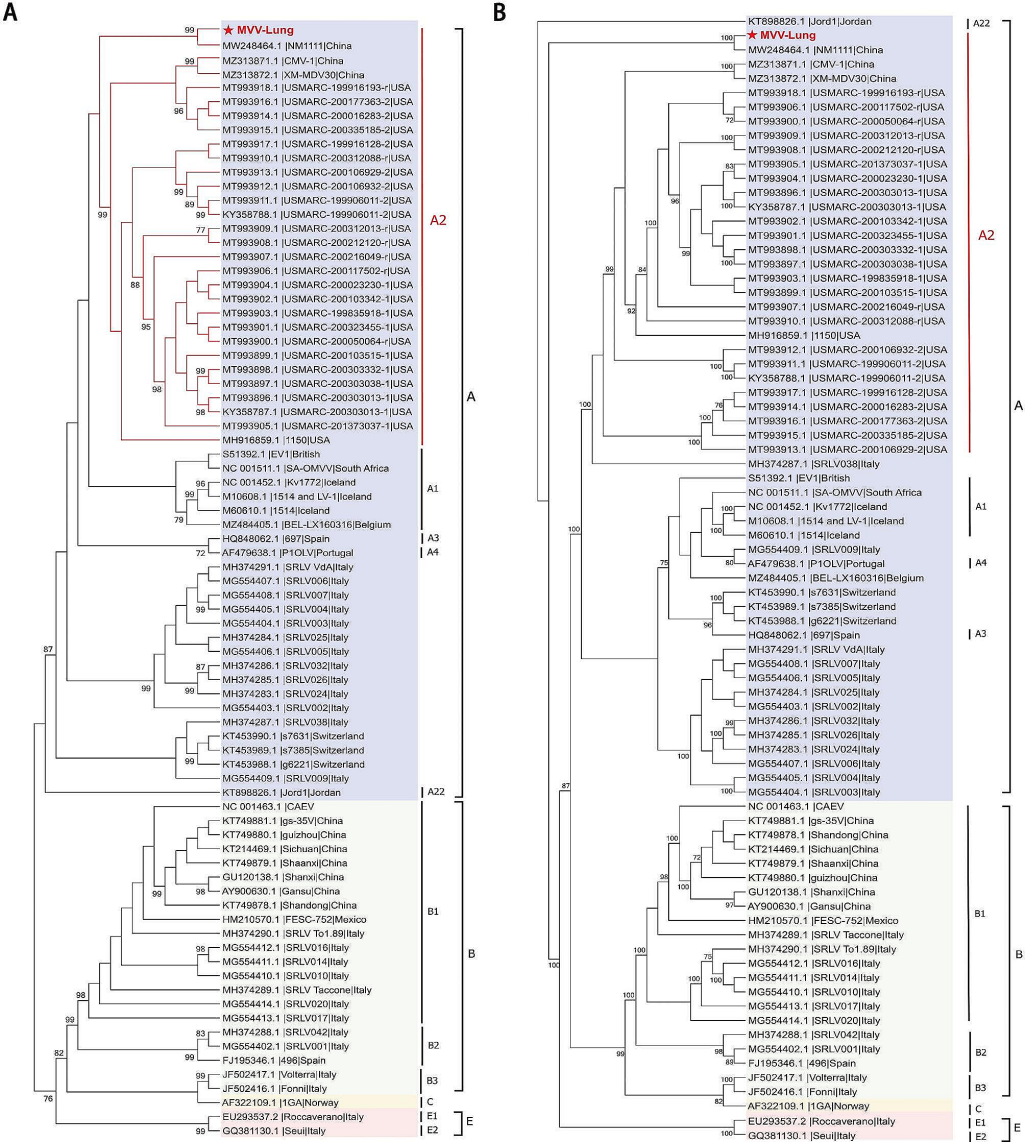
Phylogenetic trees inferred from gag and env regions. (**A**) Neighbor-Joining method phylogenetic trees based on the gag region of 79 sequences: 1 analyzed in this study (labeled by a red pentagram) and 78 whole genome sequences of SRLVs available in GenBank. (**B**) Neighbor-Joining method phylogenetic trees based on the env region of 79 sequences: 1 analyzed in this study (labeled by a red pentagram) and 78 whole genome sequences of SRLVs available in GenBank. The numbers on the nodes indicatethe percentage of bootstrap values obtained from 1000 replicates. The tree was generated using the neighbor-joining method coupled with the p-distance model and bootstrap analysis of 1,000 replicates

To confirm the genotypes of MVV-infected sheep, we used the neighbour-Joining method to map the phylogenetic trees in the gag and env regions of a total of 78 whole-genome sequences of SRLVs in GenBank, which belong to genotypes A-E. Phylogenetic trees analyses of the *gag* and *env* regions revealed that the MVV-infected sheep in this study was genetically closest to NM1111 (China) and belonged to subtype A2 (Fig. 3A and B).

### Distinct transcriptional signatures and gene expression changes in the lungs of MVV-infected sheep

**Fig. 4:**
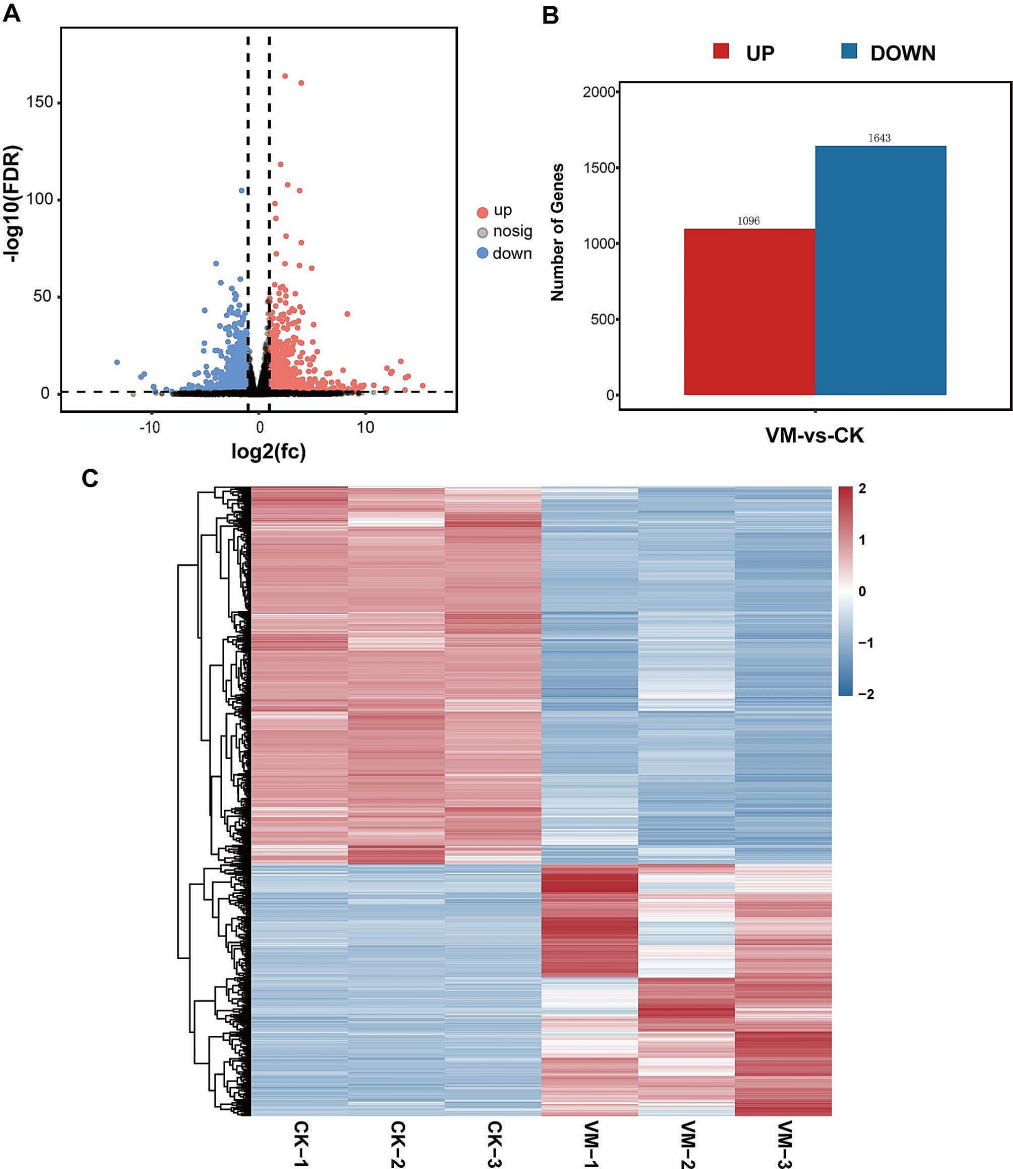
Quantitative assessment of DEGs. (**A**) Visualization of differentially expressed genes (DEGs) using volcanic plots. The log2FoldChange value is plotted on the x-axis, whereas the –log10padj value is plotted on the y-axis. Differential gene screening criteria are indicated by the black dotted line, which serves as the threshold line. (**B**) CK, control. Upregulated and downregulated DEGs are presented in the red and blue columns, respectively. Visualization of DEGs using a heatmap

We conducted an RNA-seq analysis of the pulmonary tissue between uninfected (CK) and infected (MV) samples to elucidate the transcriptomic alterations induced by MVV. The initial mean read counts for the CK and MV groups were 64,624,429 and 57,605,060, respectively. After eliminating low-quality reads, we obtained an average of 64,398,279 and 57,423,776 clean reads for the CK and MV groups, respectively. Additionally, the Q20 and Q30 percentages exceeded 98.02% and 94.19%, respectively (Supplementary Table 2), satisfying the data quality criteria. DESeq2 was used to analyze gene expression and identify DEGs in the MVV-positive and control samples, and 2,739 DEGs were identified; these included 1,643 and 1,096 downregulated and upregulated DEGs, respectively (Fig. 4A and B and Supplementary Table 3). The FPKM values were determined using mainstream hierarchical clustering, where row homogenization was achieved using the Z-score. Gene expression levels in the CK and MV samples were distinct, suggesting that MVV infection impacted gene expression in sheep lungs (Fig. 4C).

### Validation of differential mRNA expression

**Fig. 5:**
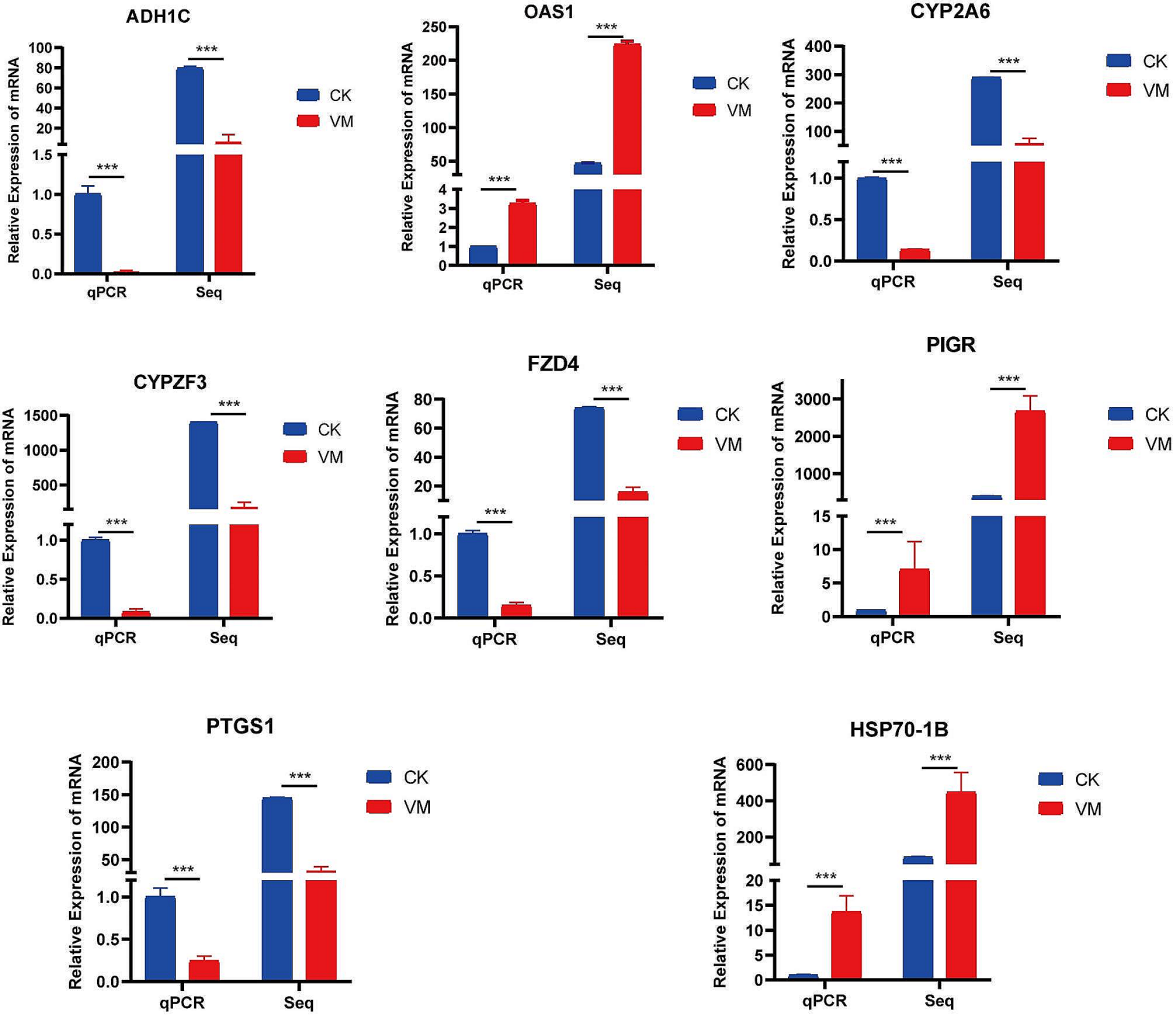
Comparison of fold-changes in differentially expressed genes between RNA-seq and quantitative reverse transcription-polymerase chain reaction. Data are presented as the mean ± standard deviation of values from three replicates. Statistical analysis was conducted using Student’s t-test, with significance indicated at *p* < 0.001

Eight genes were randomly selected and validated concurrently using RT-qPCR to verify the mRNA transcriptional levels obtained via RNA-Seq. The results revealed significant correlations among all genes across the experiments (Fig. 5), confirming the accuracy and reliability of the RNA-Seq data. Consequently, these data can be effectively used for subsequent analyses, including GO, KEGG, and Search Tool for the Retrieval of Interacting Genes/Proteins (STRING) analyses.

### GO and KEGG enrichment analyses of DEGs

**Fig. 6:**
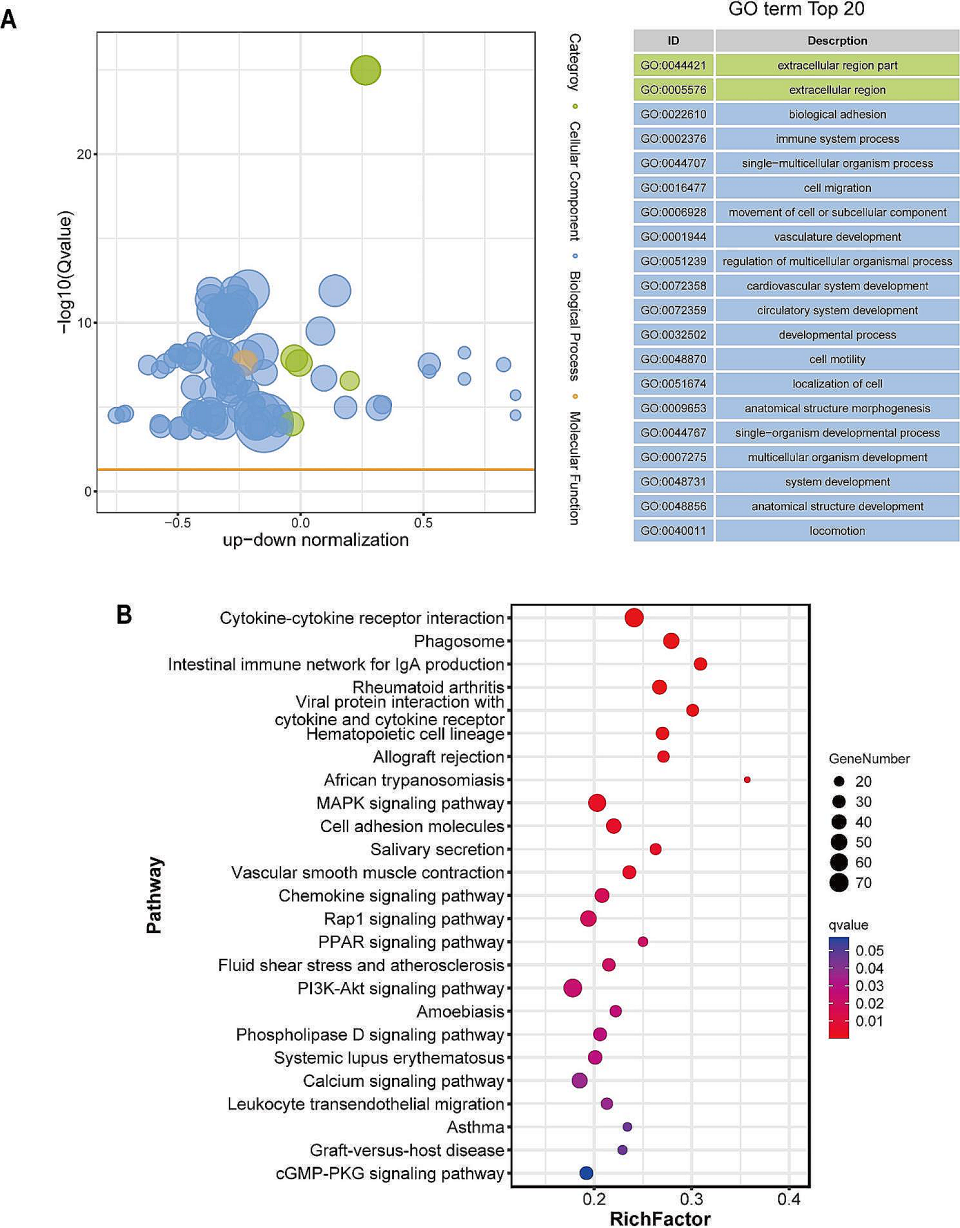
Gene ontology and Kyoto Encyclopedia of Genes and Genomes enrichment analyses of differentially expressed genes. (**A**) Gene ontology functional classification of differentially expressed genes (DEGs) according to diverse biological processes was performed. The results are visualized using a bubble plot, where the size of the bubbles represents the number of associated genes, and significantly enriched categories are labeled. (**B**) Kyoto Encyclopedia of Genes and Genomes (KEGG) enrichment analysis of the DEGs was performed. The horizontal axis of the graph indicates the proportion of DEGs annotated to a particular KEGG pathway relative to the overall number of DEGs. The y-axis represents the KEGG pathway. The number of genes annotated to the KEGG pathway is indicated by the size of the dots, and the significance of enrichment is represented by the color gradient, which ranges from red to blue

GO functional analyses were performed for the gene sets of interest, and the DEG enrichment results for the MV and CK groups were obtained considering a significance threshold of padj < 0.01. For the heatmap, terms with the highest increase and decrease in expression were selected (Supplementary Fig. 1). The results revealed that DEGs from the MV group were enriched in the biological process (BP) category, including the production of cytokines (GO: 0001816), regulation of cytokine-mediated signaling pathways (GO: 0001959), and immune system processes (GO: 0002376), and the molecular function (MF) category, including those associated with cytokine activity (GO:0005125). The top 20 elements in the MF, cellular composition, and BP categories are depicted in Fig. 6A. Furthermore, a substantial portion of these terms were directly correlated with the immune system and the protective responses against viral infections. The 20 most notable pathways were selected based on the KEGG enrichment results, as presented in Fig. 6B. These pathways were correlated with immune system functions and were consistent with the GO term patterns. In the MV group, DEGs were enriched in three signaling pathways: cytokine-cytokine receptor interaction (ko04060), intestinal immune network for IgA production (ko04672), and viral protein interaction with cytokines and cytokine receptors (ko04061). These pathways are primarily involved in inflammatory responses and correlations between viruses and host cells.

### Protein-protein interaction network construction and selection of hub genes

**Fig. 7:**
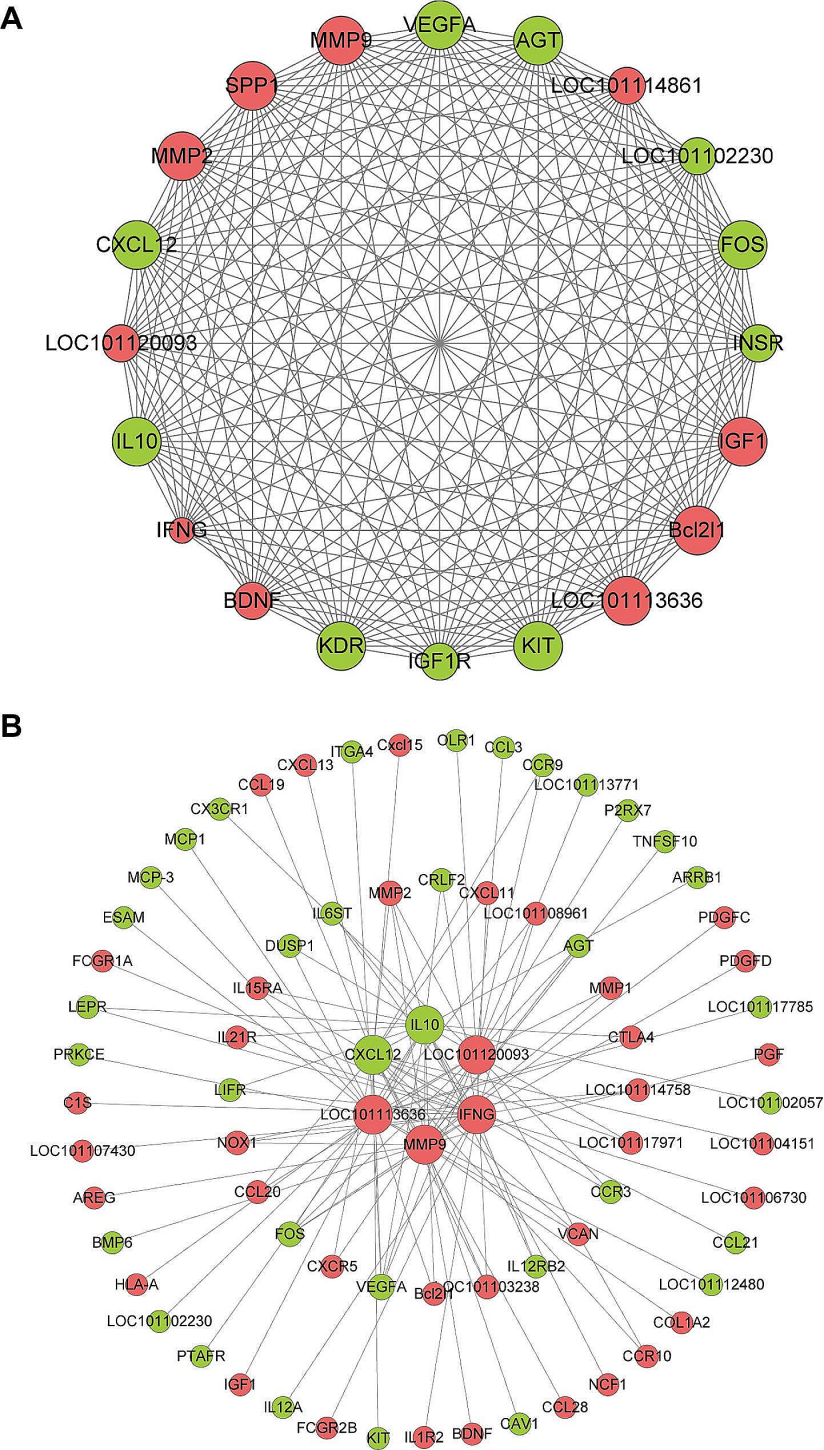
Protein-protein interaction (PPI) network analysis. (**A**) Analysis of differentially expressed genes using the STRING database. Hub genes were identified by visualizing the modules in the PPI network. The amplification of various core factors revealed significant aggregation and interaction components in the PPI network. (**B**) Cluster network of the key genes *LOC101113636*, *LOC101120093*, *MMP9*, *IFN-γ*, *CXCL12*, and *IL10*

The Cytohubba plugin of Cytoscape 3.9.1 was utilized to identify hub genes in the protein-protein interaction (PPI) network consisting of 2,739 nodes and 83,923 edges, which was generated from STRING. The top 20 hub genes with the highest connectivity were S*PP1, LOC101113636, LOC101120093, MMP9, MMP2, LOC101114861, Bcl2l1, IGF1, BDNF, IFN-γ, FOS, KIT, LOC101102230, AGT, KDR, IGF1R, INSR, VEGFA, CXCL12*, and *IL10* (Fig. 7A).

We further expanded the cluster to identify the genes that interacted. In this smaller section, 69 genes were clustered together, with *LOC101113636, LOC101120093, MMP9, IFN-γ, CXCL12*, and *IL10* as the central genes (Fig. 7B). A higher proportion of overexpressed genes formed groups, including those for IFN-γ and cytokines, such as *CXCL11, IL21R, IL15RA, IL1R2, FCGR1A, FCGR2*, and *NOX1*. MVV infection upregulated genes related to antiviral defense pathways but downregulated genes associated with circadian entrainment pathways.

### Expression of genes related to the immune response

**Fig. 8:**
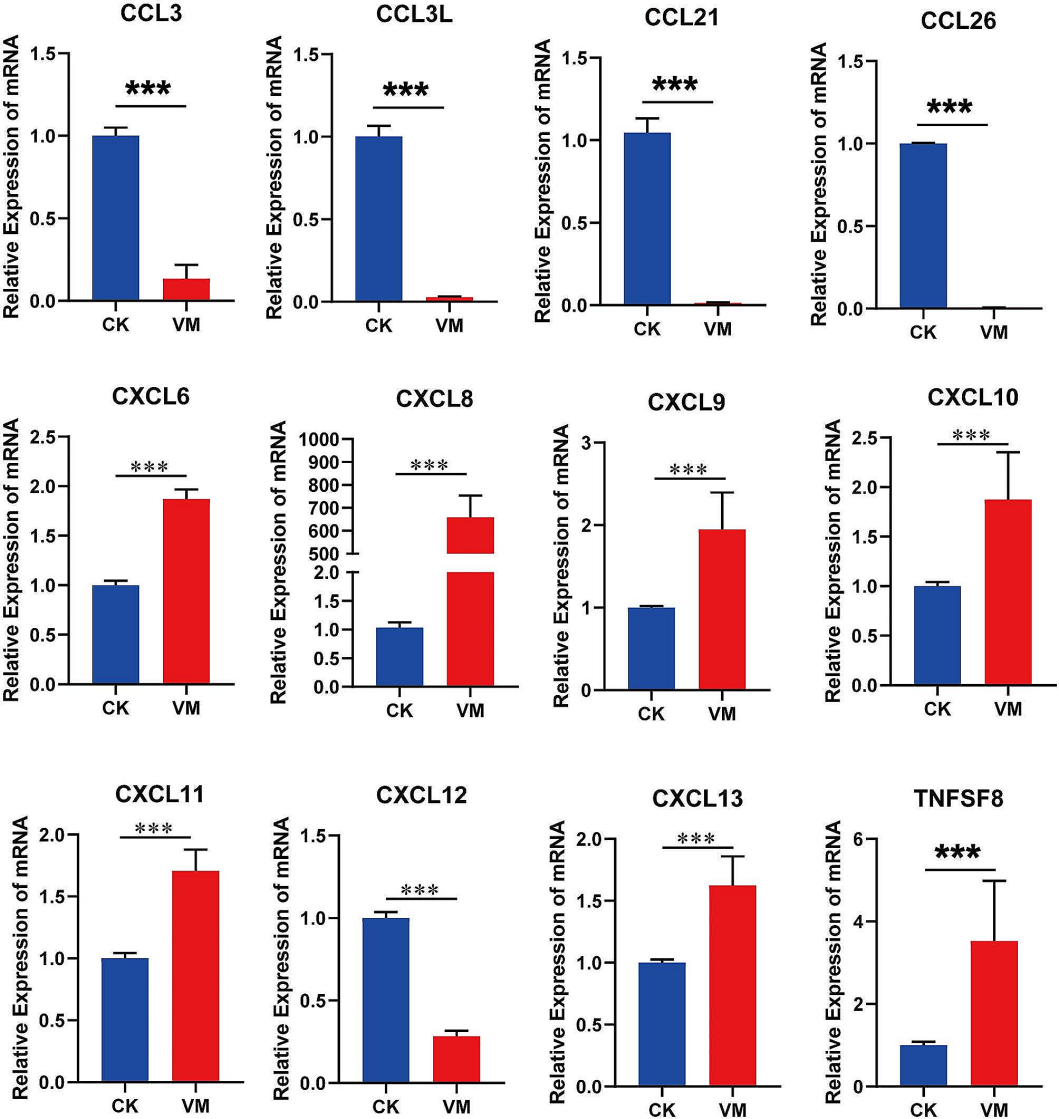
Expression of chemokine genes in the cytokine-cytokine receptor interaction pathway

**Fig. 9:**
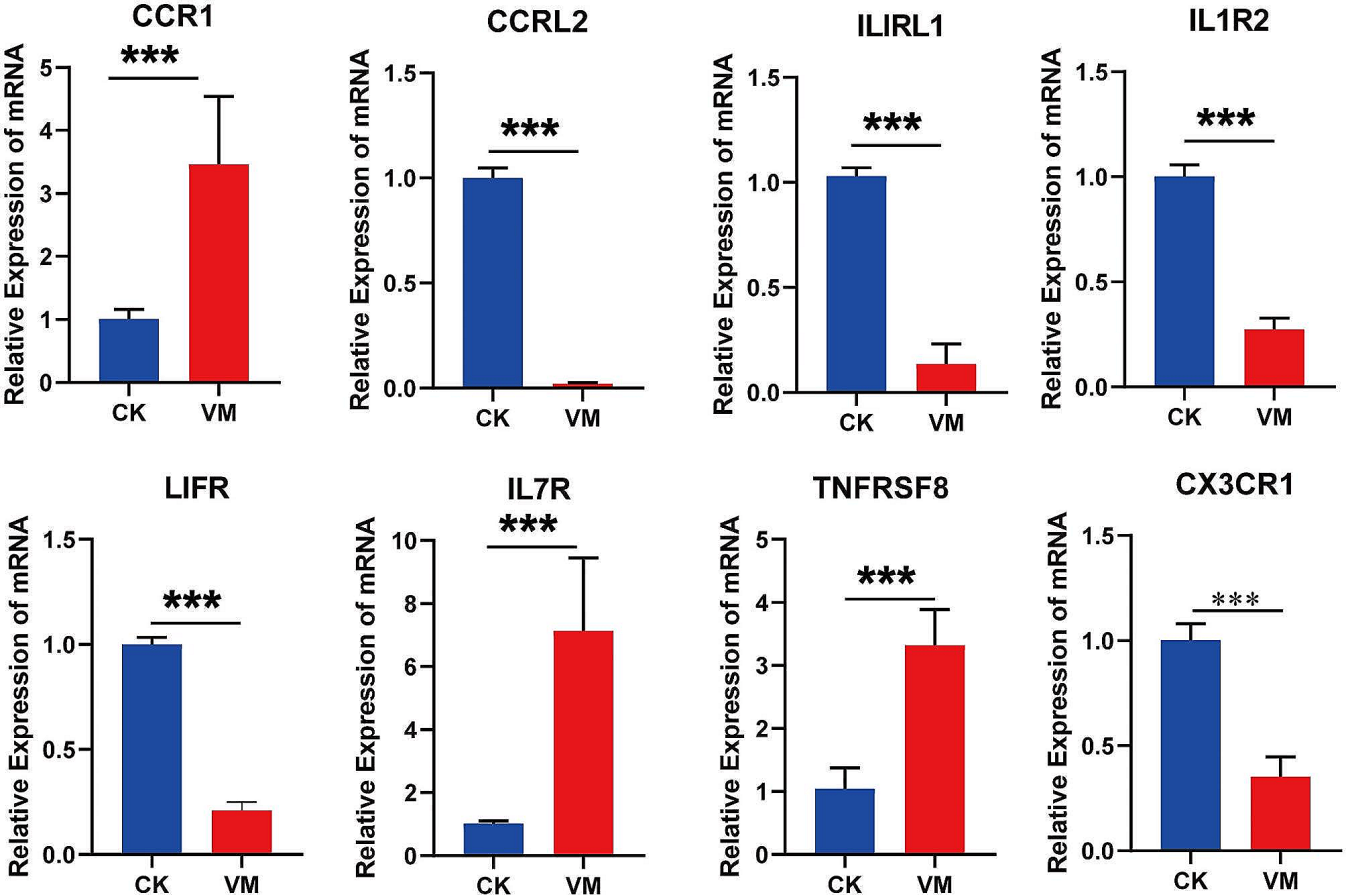
Expression of chemokine ligand and receptor genes in the cytokine-cytokine receptor interaction pathway

**Fig. 10:**
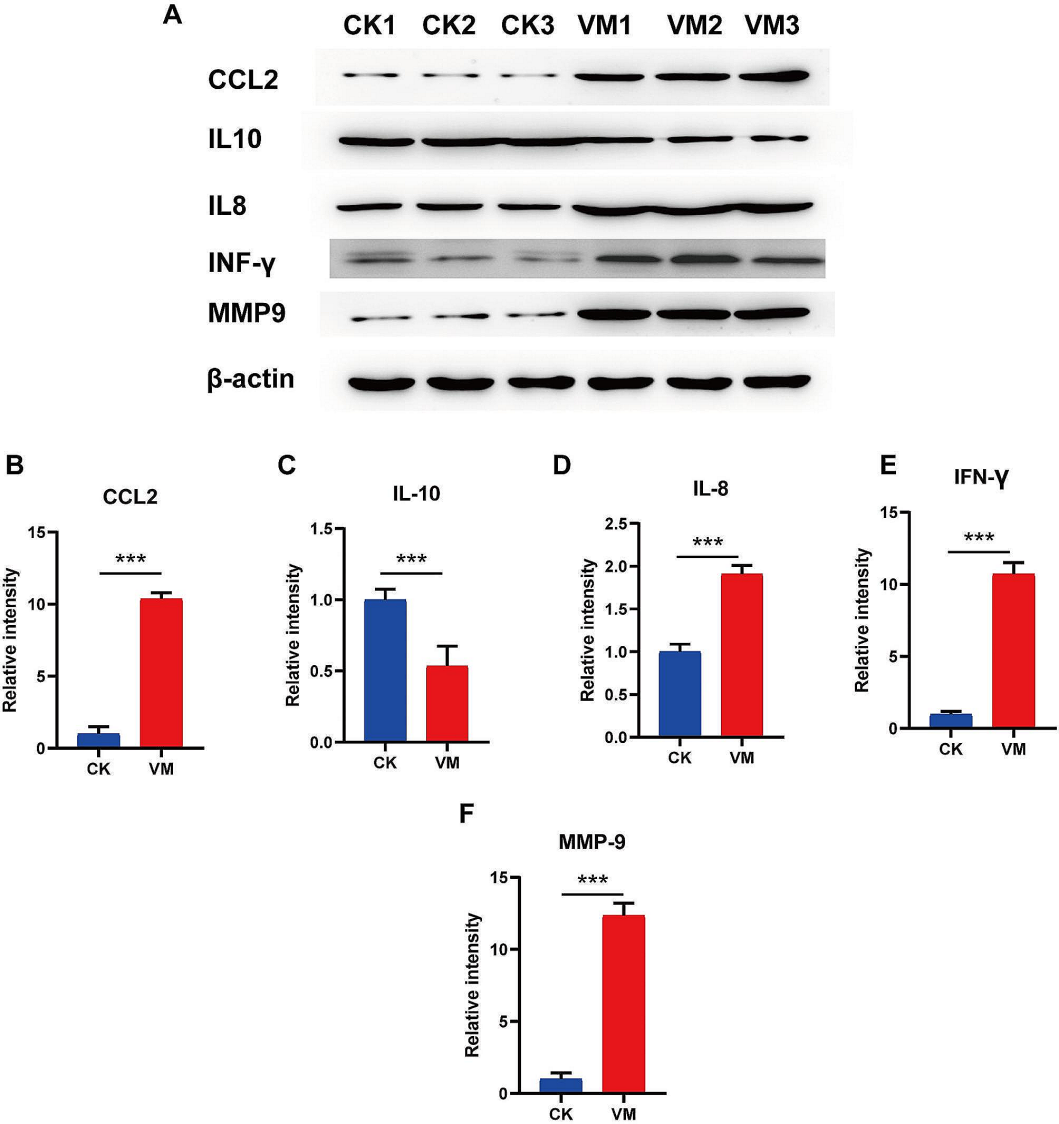
Relative protein expression of significantly altered inflammatory cytokines. (**A**) CCL2, IFN-γ, IL-8, IL-10, and MMP9 expression in the lungs of MVV-infected sheep confirmed through western blotting. (**B**–**F**) Quantitative analysis of CCL2, IFN*-γ*, IL-8, IL-10, and MMP9 expression

Cytokines play a crucial role in MVV-induced pathogenesis by regulating immune activation in target organ lesions, where certain cytokines promote lesion formation. Therefore we analyzed the expression of important genes involved in the cytokine-cytokine receptor interaction (ko04060) and viral protein interaction with cytokine and cytokine receptor (ko04061) signaling pathways using RT-qPCR. In the lungs of MVV-infected sheep, the expression *of CCL3, CCL21, CCL26, CCL3L, CCRL2, CXCL12, ILIRL1, LIFR, IL1R2*, and *CX3CR1* significantly decreased while that *of CXCL13, CXCL6, CXCL11, CCR1, CXCL8, CXCL9, CXCL10, IL7R, TNFRSF8*, and *TNFSF8* significantly increased (Figs. 8 and 9). Furthermore, we analyzed the protein expression of CCL2, IFN-γ, IL-8, IL-10, and MMP9 via WB and found that CCL2, IFN-γ, IL-8, and MMP9 were greatly upregulated in the pulmonary region of MVV-infected sheep (Fig. 10), whereas IL-10 was notably downregulated (Fig. 10). These chemotactic cytokines regulate the movement of other cells in response to chemical stimuli (chemotaxis). The significant alterations in gene expression observed in the lungs of MVV-infected sheep suggest that these genes play a significant role in the development of lung pathologies.

### MVV infection results in infiltration and proinflammatory activation of macrophages

**Fig. 11:**
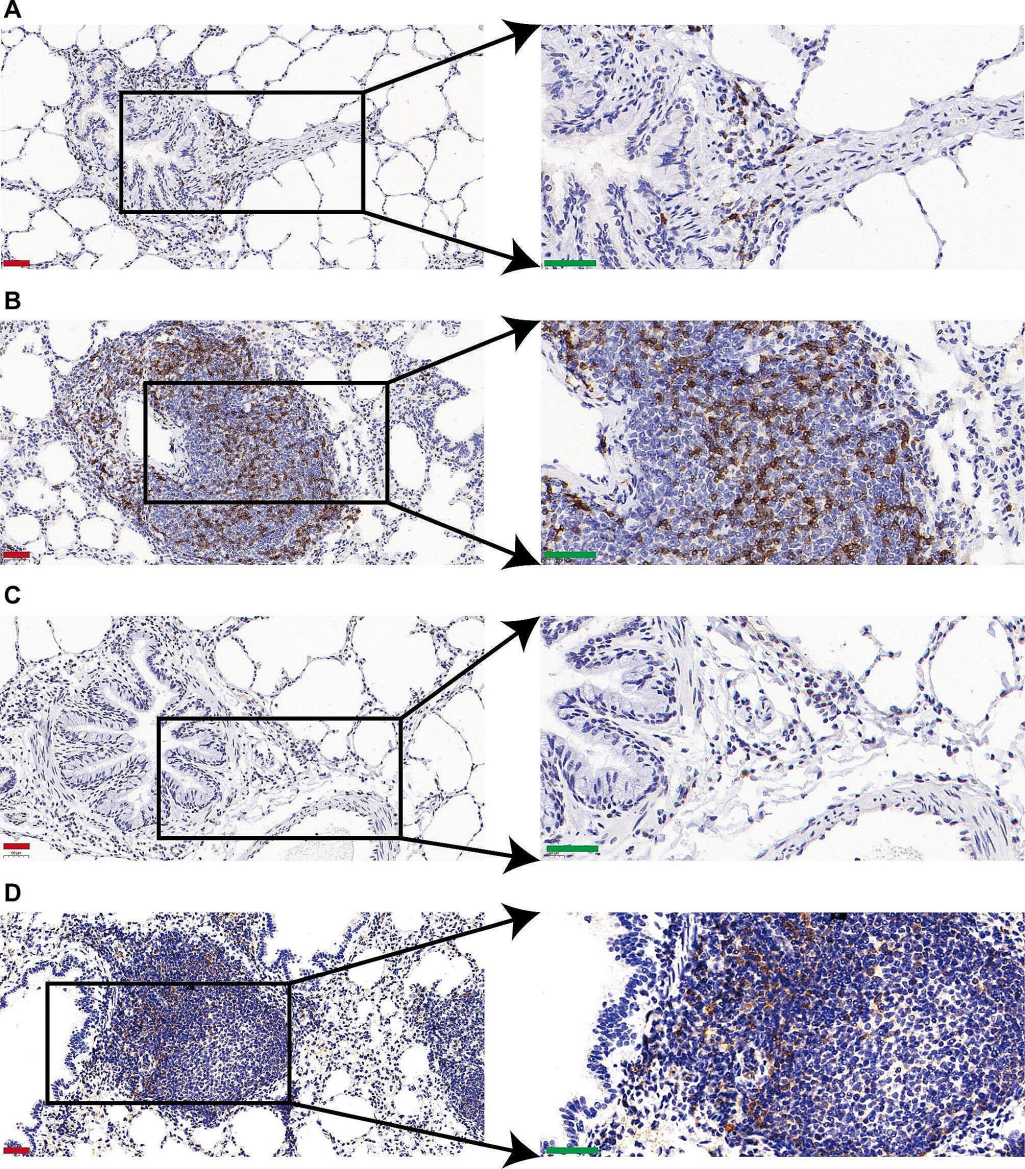
CD4 and CD8 markers used for immunostaining of immune cells in lung sections. (**A**) Control samples for CD4 immunostaining. (**B**) CD4 immunostaining of lungs from Maedi-visna virus (MVV)-infected sheep. (**C**) Control samples for CD8 immunostaining. (**D**) CD8 immunostaining of lungs from MVV-infected sheep (red bar, 50 μm and green bar, 40 μm)

**Fig. 12:**
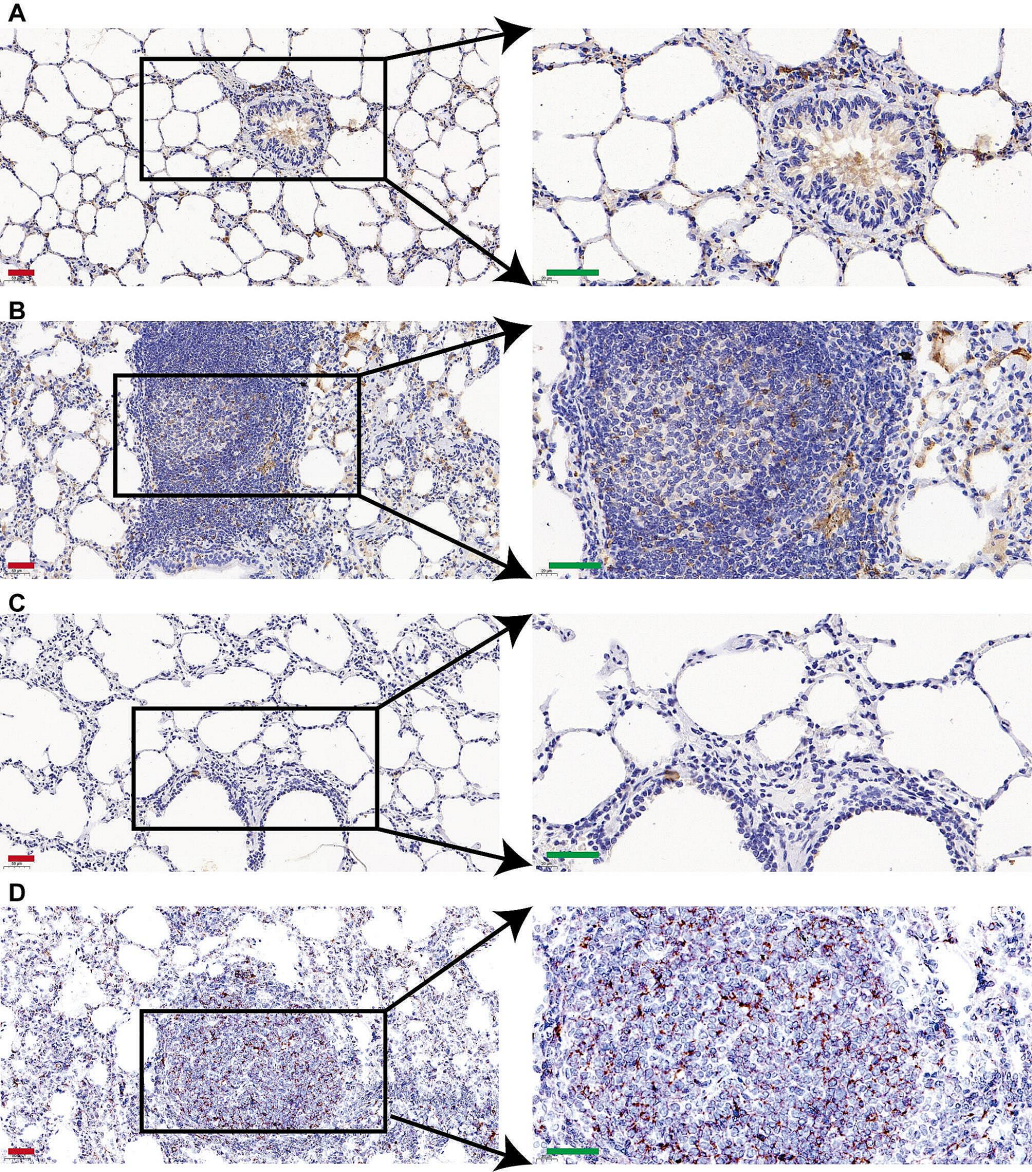
Immunostaining of CD68-positive macrophages and CD19‐positive B cells infiltrating the lung. (**A**) Control samples for CD68 immunostaining. (**B**) CD68 immunostaining of lungs from Maedi-visna virus (MVV)-infected sheep. (**C**) Control samples for CD19 immunostaining. (**D**) CD19 immunostaining of lungs from MVV-infected sheep (red bar, 50 μm; green bar, 40 μm)

Immunohistochemical analysis was conducted on the lungs of MVV-infected sheep to detect CD4^+^ and CD8^+^ T cells, B cells, and macrophages (Figs. 11 and 12). Immunostaining was observed in the cytoplasm and membranes of mononuclear cells within the inflamed area and the bronchus-associated lymphoid tissue region of the lungs. Figure 11A–D shows that the perivascular cuffs in the lungs (indicating MVV infection) consisted of CD4 + T cells and CD8 + lymphocytes. Moderate quantities of macrophages were present primarily in the central regions of lesions within the lungs and found near mildly affected blood vessels at the periphery of the lesion (Fig. 12A and B). The germinal centers of well-developed lymphoid follicles were mostly composed of B cells, which were rarely detected elsewhere (Fig. 12C and D), suggesting that lymphocytes, monocytes, macrophages, and B cells invade the interalveolar septa, leading to the thickening and hyperplasia in the alveolar walls, promoting the development of pulmonary diseases.

## Discussion

The current study employed next-generation sequencing to examine mRNA expression patterns in the lung tissues of healthy and MVV-infected sheep. Using RNA-seq analysis, we identified several host cellular factors associated with MVV infection. Some of these factors may directly contribute to MVV infection, whereas others may have an unknown or indirect role in MVV infection. Various biological processes are altered by interactions between the host and the virus owing to the capacity of this virus to use the mechanism of the host for replication and the response of the host to resist infection.

MVV infections in sheep are difficult to diagnose considering clinical manifestations, and several months to years may lapse before sheep infected with MVV develop fatal pneumonia or encephalomyelitis [6, 42]. Therefore, the disease is diagnosed by identifying distinct abnormalities via visual examinations and microscopy and assessing the medical history of the sheep, and, serological and molecular tests are used to confirm infection [43–47]. Various techniques, including histopathological examination, ELISA, IF assay, immunohistochemistry (IHC) staining, PCR, and TEM, were employed to assess MV disease in sheep, and all PCR-positive lung samples exhibited MV virions inside the macrophage.

RNA-seq data analysis revealed significant DEGs in healthy and MVV-infected lung tissues. Furthermore, 2,739 DEGs were identified, including 1,643 and 1,096 downregulated and upregulated DEGs, respectively. By combining the RNA-Seq and RT-qPCR data, we confirmed that the results obtained from RNA-Seq were accurate and reliable. Furthermore, the polymeric immunoglobulin receptor, 2′, 5′-oligoadenylate synthetase 1, and heat shock protein 70 kDa protein 1B genes were highly expressed in MVV-infected lung tissue.

We performed GO and KEGG pathway analyses to annotate alterations in gene expression levels following MVV infection. The GO analysis indicated that the host mounted a robust immune response against MVV, which involved the generation of cytokines (GO: 0001816), control of cytokine-mediated signaling pathways (GO: 0001959), and promotion of immune system development (GO: 0002376). A comparison of lung tissue from MVV-infected sheep with that from a healthy sheep revealed that the three pathways were significantly enriched in the infected sheep. The PPI networks of DEGs revealed that immune-related genes, including those encoding cytokines and interferon regulatory factors, were highly connected hub genes.

The initial immune is the primary barrier against pathogen invasion [48]. Inflammation-inducing cytokines are vital in triggering innate immune cell activation, complement activation, and specific immune responses. The findings of our study indicated that the DEGs modified when sheep alveolar macrophages were infected with MVV were highly enriched in the KEGG pathway related to the interaction between cytokines and cytokine receptors. Of the 70 DEGs participating in this pathway, 30 and 40 genes exhibited notable downregulation and significant upregulation, respectively. Chemokines are secreted proteins that induce cell movement, particularly among leukocytes [49]. The CXCL family, a part of the chemokine family, considerably impacts the manifestation of diseases associated with chemokines [50]. In the present study, CXCL6, CXCL9, CXCL10, CXCL11, and CXCL13 were significantly upregulated, whereas CXCL12 was notably downregulated in the lungs of MVV-infected sheep. Several studies have shown that IFN-γ and IFN-γ-inducible chemokine ligand (CXCL)9, -10, and − 11 attract inflammation and infiltrates to the parenchyma because they cannot regulate the infection process [51–53]. Chronic hepatitis C caused by the hepatitis C virus and intrahepatic interferon-gamma (IFN-γ), which causes increases in CXCL10 production by the sinusoidal endothelium and hepatocytes, subsequently attracting T-cells expressing CXCR3 to the liver. Thus, CXCL10 plays a vital role in the progression of necroinflammation and fibrosis. Additionally, in the context of HIV infection, CXCL10 levels in plasma are strongly correlated with CD4 + T cell counts and viral loads [53, 54]. However, further studies should determine the role of CXCL10 in the pathogenesis of MVV and its potential as a biomarker. However, we observed a substantial increase in IFN-γ levels in the lungs of MVV-infected sheep through immunoblotting. Previous studies have indicated that inflammatory chemokines, such as CCL2, significantly impact the immune response to viral infections by increasing the cytotoxicity of the inflammatory immune cells and promoting the release of antiviral mediators. Contrastingly, the overproduction of secretions by these chemokines following infection leads to hyperinflammation [55]. In the current study, CCL2 protein levels were notably elevated in the lungs of MVV-infected sheep. Moreover, the concentrations of IL-8 and MMP9 proteins were markedly increased, whereas IL-10 levels were notably reduced in the lungs of MVV-infected sheep. These findings imply the involvement of cytokines in initiating immune responses against microbial agents, such as MVV, and the subsequent development of immune-related disorders. However, further studies are required to reveal the complex molecular mechanisms underlying this phenomenon.

Considering the significant role of cell-mediated immunity in determining the severity of respiratory diseases, IHC analyses of cellular populations in each lesion type are important for understanding the pathogenesis of a disease. In this study, lung damage was identified considering the abundance of infiltrating T, CD4^+^ T, CD8^+^ T, and B lymphocytes and macrophages, consistent with the results of previous studies on the fundamental causes of diseases [4, 31, 32, 56–61]. The pathogenesis of various diseases may be affected by exacerbated secretions by proinflammatory cytokines or the cytotoxic activity of these cells.

**Fig. 13:**
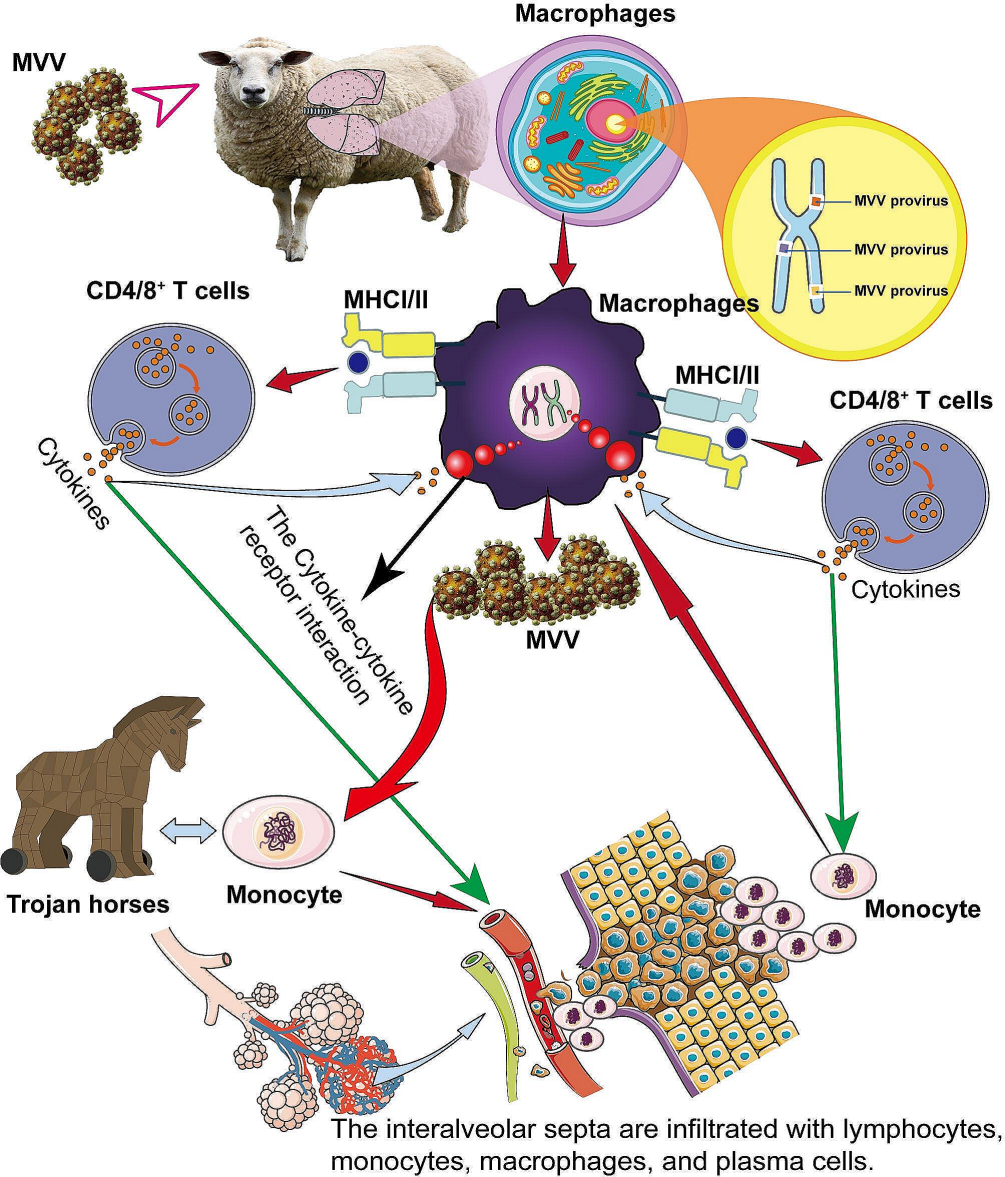
Schematic diagram of the pathogenic mechanism of ovine progressive pneumonia caused by Maedi-visna virus (MVV). MVV infection begins with the presence of macrophages in the lung mucosa. Antigen presenting cells, also known as macrophages, process viral proteins into antigens. T lymphocytes recognize the viral protein-major histocompatibility complex, triggering the synthesis of interferons I and II. This attracts additional inflammatory cells to the core, thereby supporting ongoing viral replication and persistent inflammation. CD4/8 + T cells undergo activation and differentiation into Th1 and Th2 effector cells and various subpopulations, such as Tfh cells. Cytotoxic T cells secrete cytokines such as INF-γ and chemokines to recruit immune cells. Continuous immune activation occurs in MVV-induced lesions, releasing cytokines that stimulate the transformation of monocytes into macrophages. The distinction between macrophages enables the ongoing display of viral antigens to T cells. Consequently, the stimulated T cells generate additional cytokines, thereby establishing a detrimental cycle

In addition to discussing the findings of this study, this section provides a concise summary of the literature on the development and process of ovine progressive pneumonia (Fig. 13). The beginning of infection is recognized by the recruitment of macrophages to the lung mucosa. The monocyte/macrophage lineage links innate and adaptive immunity against MVV. Antigen-presenting cells process the viral proteins as antigens and infected macrophages have viral proteins on their surface that are largely connected to the major histocompatibility complex (MHC). T lymphocytes recognize the viral protein MHC, triggering the synthesis of interferons I and II, attracting additional inflammatory cells to the core, thereby supporting ongoing viral replication and persistent inflammation. Inflammatory cells release inflammatory cytokines that may stimulate or prevent monocyte maturation in macrophages, thereby controlling viral replication [32, 62]. Cell-mediated immune responses against MVV are crucial for defense against viral infections, particularly through the action of cytotoxic T cells. Infected monocytes and macrophages activate specific cytotoxic T cells, which may hinder viral replication. Nevertheless, it is crucial to acknowledge that these activated cells can play a role in the formation of lesions by generating cytokines or exerting cytotoxic impacts. Nonetheless, continuous immune activation occurs in MVV-induced lesions and cytokines are released, stimulating the transformation of monocytes into macrophages. The distinction between macrophages enables the ongoing display of viral antigens in T cells [17]. Consequently, the stimulated T cells generate an increased amount of cytokines, leading to the formation of a detrimental cycle (Fig. 13). Monocytes in the bloodstream are susceptible to infection, albeit with minimal viral transcription. However, upon differentiation into tissue macrophages, viral replication becomes active, leading to substantial levels of viral transcription and the subsequent production of viral proteins. Consequently, immature macrophages serve as “Trojan horses” that surreptitiously disseminate virus-infected cells that evade detection or eradication by the immune system, thereby infiltrating various tissues [63, 64]. Subsequently, these cells undergo maturation within the tissues, facilitating viral replication.

## Conclusions

Our study reveals that the transcriptome profile of sheep changes after MVV infection. A range of immune response mechanisms were observed, including molecular and cellular defense mechanisms, cytokine-cytokine receptor interactions, and viral protein interactions with cytokines and cytokine receptors, involving 2,739 DEGs. Thus, the pathogenesis of MVV is significantly influenced by inflammatory cytokines. This study revealed novel genes and pathways associated with MVV infection and development. Discovering new genes related to viral infections and pathogenesis, enhances our understanding on how MVV causes infection and disease.

### Electronic supplementary material

Below is the link to the electronic supplementary material.

Supplementary Material 1

Supplementary Material 2

Supplementary Material 3

Supplementary Material 4

Supplementary Material 5

## Data Availability

The datasets generated and/or analyzed during the current study are available in the National Center for Biotechnology Information (NCBI) repository (SubmissionID: SUB14261107; BioProject ID: PRJNA1079058). https://www.ncbi.nlm.nih.gov/bioproject/?term=PRJNA1079058.
