# Supplementary material for: Differential gene expression and immune cell infiltration in maedi-visna virus-infected lung tissues
Source: BMC Genomics. 2024 May 30;25:534. doi: 10.1186/s12864-024-10448-2 (PMC11141007; doi:10.1186/s12864-024-10448-2)
Supplement: Supplementary file 1 — Supplementary Material 1 [file 12864_2024_10448_MOESM1_ESM.docx]

**Supplementary Information**

**Supplementary Figure 1**

Heat map for GO enrichment analysis. The figure presents the number of GO terms in the differential gene background and Q. The smaller the Q, the redder the color.

**Supplementary Table 1**

Primer sequences and real-time polymerase chain reaction amplification parameters.

**Supplementary Table 2**

Overview of sequencing. Prior to the analysis of raw data, data filtering should be performed on the raw data to reduce interference from invalid data. To obtain clean reads, we used fastp to control the quality of the downlinked raw reads.

**Supplementary Table 3**

Overall information on differentially expressed genes. Using DESeq2 software analysis, significant differences were determined when a gene had a false discovery rate of 0.05 and log2FC >1.

**Additional File 1**





Additional File l: **PCR amplification of MV provirus.** The expression is checked in different sheep, Lane M presents 500 bp molecular weight markers; Lanes 1–3 present lung tissue samples from three MVV-infected sheep; Lanes 4–6 present lung tissue samples from three healthy sheep; and Lane 7 presents the negative control.

(A) CCL2 (~8.8 kDa)


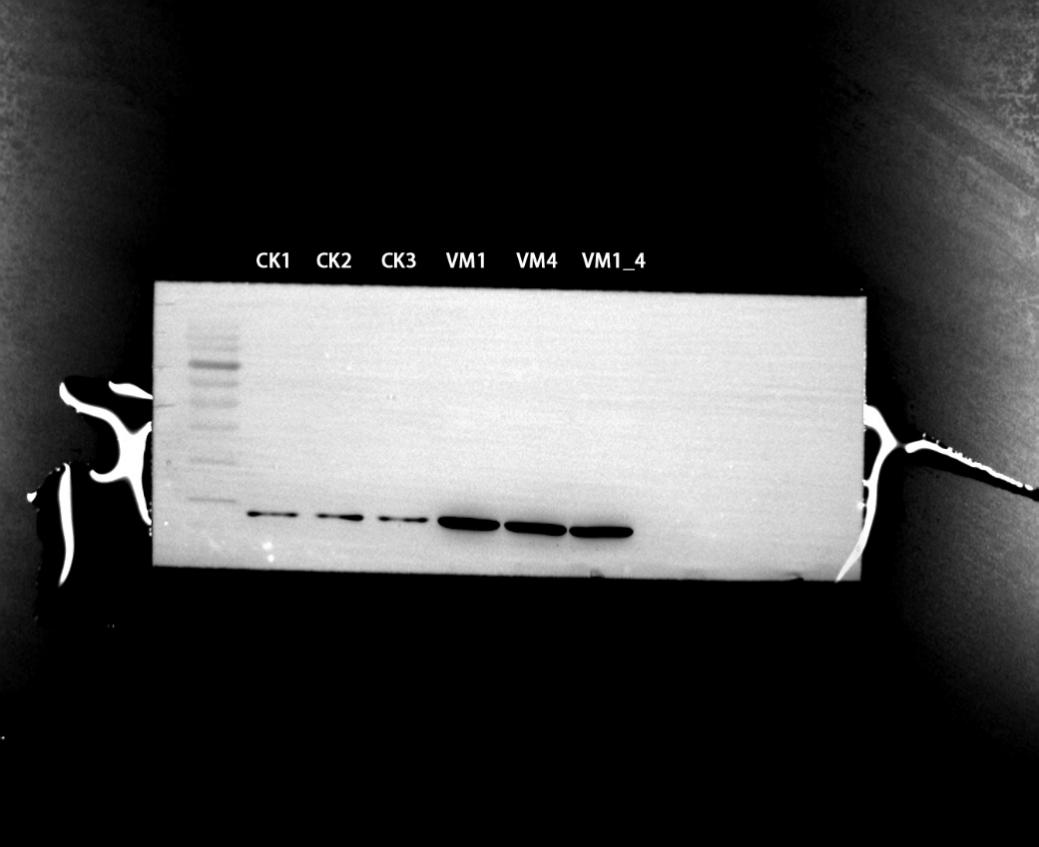


(B) IL-8 (~11 kDa)





(C) IL-10 (~19 kDa)


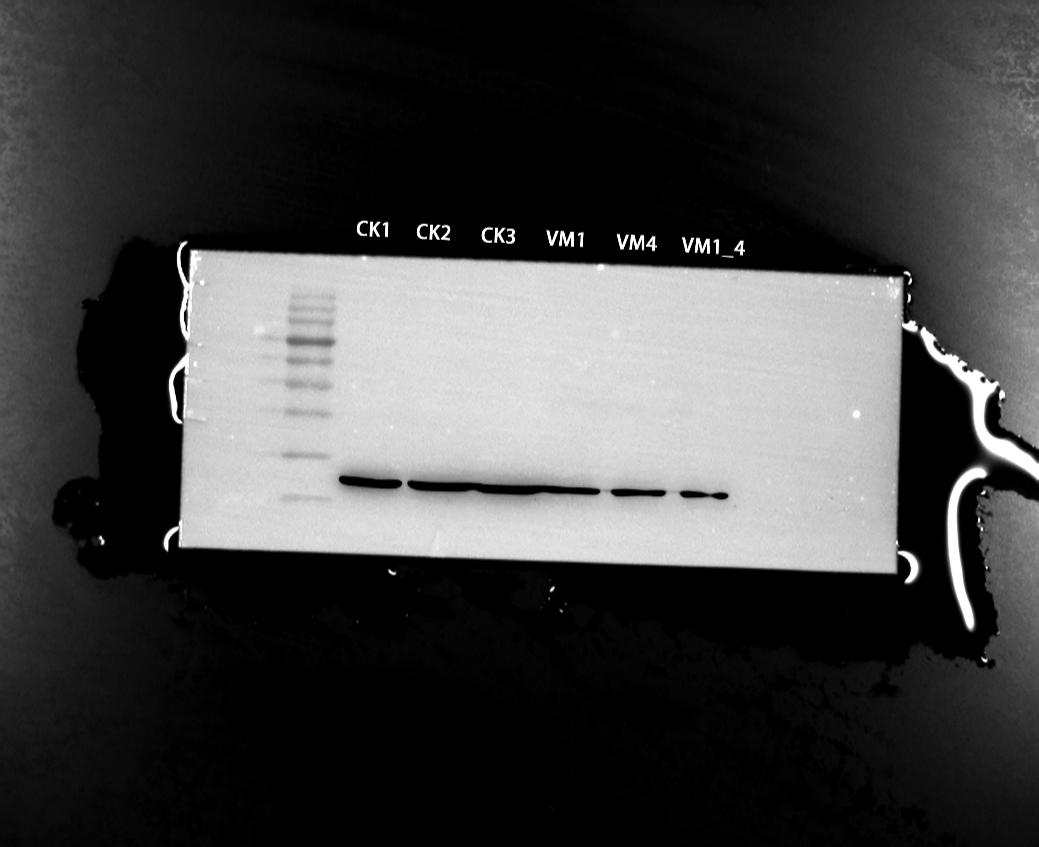


(D) MMP9 (~78 kDa)


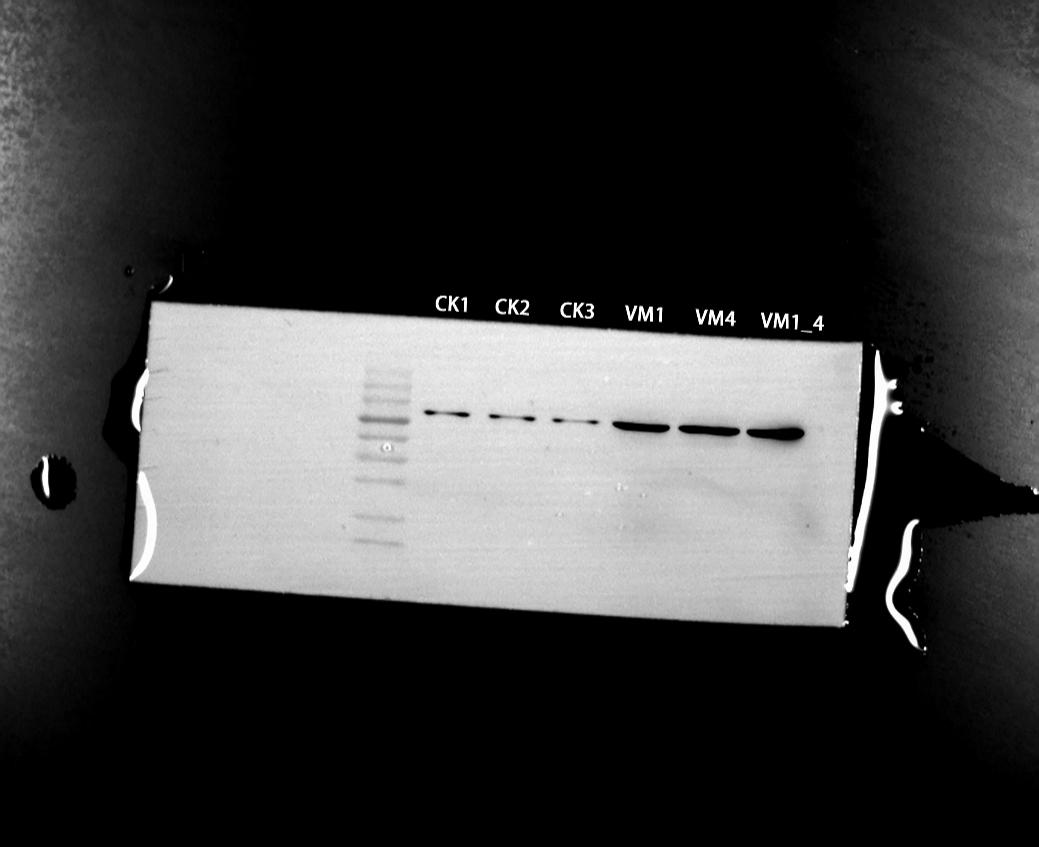


(E) IFN-γ





(F) β-actin (~42 kDa)





Additional File 2: Western blot analysis of protein expression in sheep lung tissue, Full blot image of CCL2 protein in sheep lung tissue (A). Full blot image of IL-8 protein in sheep lung tissue (B). Full blot image of IL-10 protein in sheep lung tissue (C). Full blot image of MMP9 protein in sheep lung tissue (D). Full blot image of IFN-γ protein in sheep lung tissue (E). Full blot image of β-actin protein in sheep lung tissue (F).
