# Supplementary figures and images for: Differential gene expression and immune cell infiltration in maedi-visna virus-infected lung tissues
Source: BMC Genomics. 2024 May 30;25:534. doi: 10.1186/s12864-024-10448-2 (PMC11141007; doi:10.1186/s12864-024-10448-2)

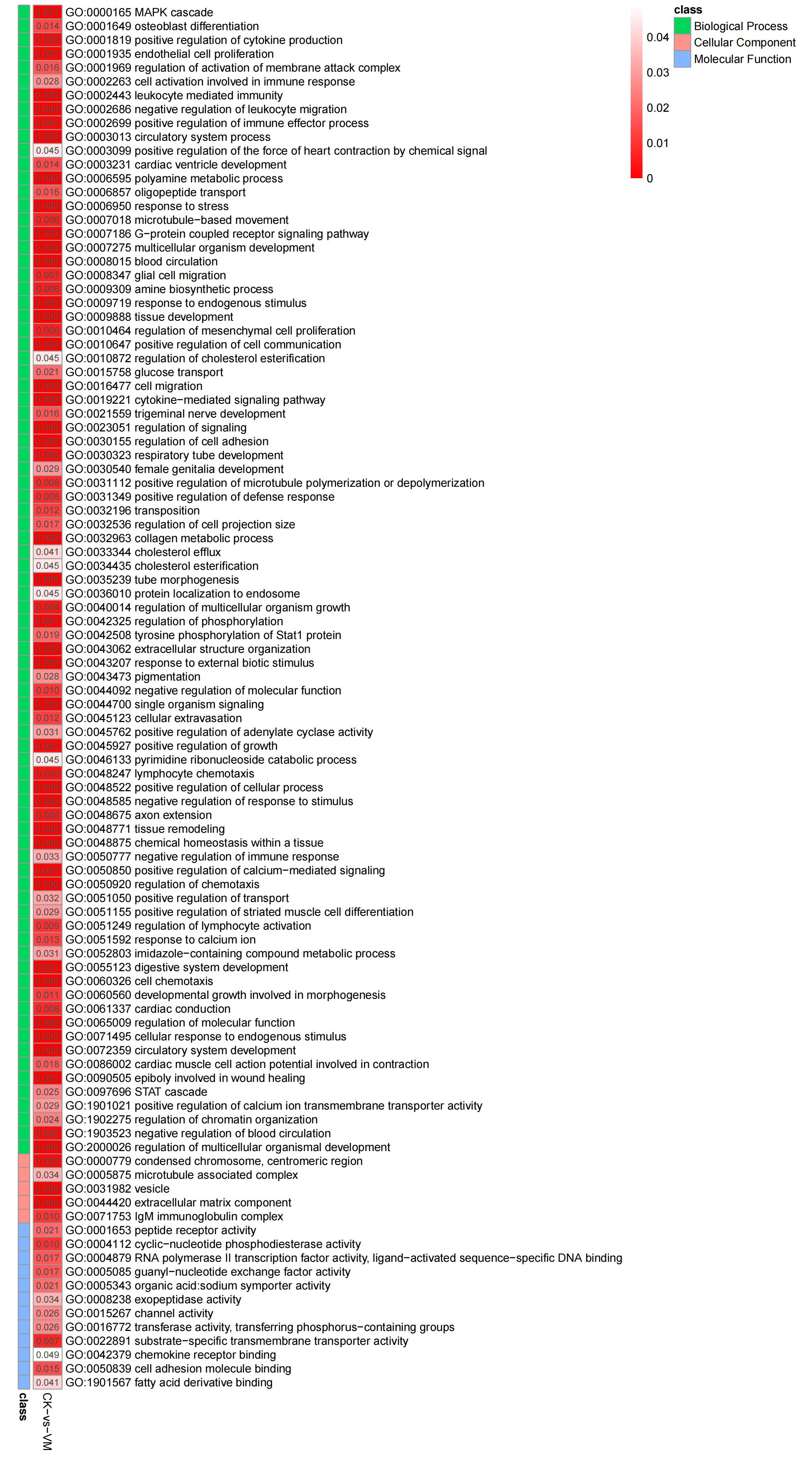

Supplement: Supplementary file 2 — Supplementary Material 2 [file 12864_2024_10448_MOESM2_ESM.jpg]
