# Supplementary material for: Differential gene expression and immune cell infiltration in maedi-visna virus-infected lung tissues
Source: BMC Genomics. 2024 May 30;25:534. doi: 10.1186/s12864-024-10448-2 (PMC11141007; doi:10.1186/s12864-024-10448-2)
Supplement: Supplementary file 3 — Supplementary Material 3 [file 12864_2024_10448_MOESM3_ESM.docx]

Supplementary Table S1 Primers sequences and real-time PCR amplification parameters.

| Genes | Accession No. | Primer (5'-3') | Product size/bp | Annealing temperature/℃ |
| --- | --- | --- | --- | --- |
| β-actin | NM_001009784.3 | GTCACCAACTGGGACGACA  AGGCGTACAGGGACAGCA | 208 | 60.0 |
| PIGR | XM_004013573.4 | CATCGACTCCACCGGGTATG  TCGGTTGATGACAACGCTGA | 100 | 60.0 |
| CYP2A6 | XM_004015267.5 | AGCAGCAGGCCTTTAAGGAG  TCAAACTTGGGCTGACGGTT | 331 | 60.0 |
| CYP2F3 | XM_027978286.2 | TTCGGCATGGGGAAGAGAAC  ATCGTCGTCGTAGTCGAAGC | 177 | 60.0 |
| PTGS1 | [NM_001009476.1](https://www.ncbi.nlm.nih.gov/entrez/viewer.fcgi?db=nucleotide&id=57164168) | ATCCACTTTCTGCTGACGCA  GGGGATAAGGTTGGAACGCA | 111 | 60.0 |
| FZD4 | [XM_027959902.2](https://www.ncbi.nlm.nih.gov/entrez/viewer.fcgi?db=nucleotide&id=2062847348) | ACCTGCGTGATTGCCTGTTA  CCACGTGTGCAGAGTTTTGG | 168 | 60.0 |
| ADH1C | XM_004009680.4 | GGTTATGGGTCTGCGGTCAA  TCAAGCCGACCGATGACTTC | 284 | 60.0 |
| OAS1 | [XM_012097882.3](https://www.ncbi.nlm.nih.gov/entrez/viewer.fcgi?db=nucleotide&id=2062802941) | TCAGCTTTGTGCTGAGGTCC  TCCAAGCTGCTCCTTACACAG | 278 | 60.0 |
| HSP70-1B | [XM_042236765.1](https://www.ncbi.nlm.nih.gov/entrez/viewer.fcgi?db=nucleotide&id=2062838307) | TCTCGGAGTCGAAAAAGCGG  GTCGTTGATCACGCGGAAAG | 319 | 60.0 |
| CXCL9 | XM_004009924.6 | GGAGTTCAAGGAATCCCAGCA  ACAAGTAGGGCTTGGAGCAA | 120 | 60.0 |
| CXCL10 | NM_001009191 | CCTCGAACACAGAAAGAGGCA  TCCCCCTGCAGGAGTAGTAG | 183 | 60.0 |
| ILIRL1 | [XM_042246362.1](https://www.ncbi.nlm.nih.gov/entrez/viewer.fcgi?db=nucleotide&id=2062877160) | GCAGAATACAAGCGACGCAG  TTCAAGAACGTCGGGCAGAA | 434 | 60.0 |
| IL1R2 | [XM_042246374.1](https://www.ncbi.nlm.nih.gov/entrez/viewer.fcgi?db=nucleotide&id=2062877214) | GCATAGAGGACGCCTACCAC  CAGTGGCACCTCAACGTAGT | 92 | 60.0 |
| IL7R | [XM_042233877.1](https://www.ncbi.nlm.nih.gov/entrez/viewer.fcgi?db=nucleotide&id=2062798888) | GGCTCTGATGGTCACTCTGG  CACTAGAGGTCTGCCCTTGC | 442 | 60.0 |
| CCRL2 | [XM_042236372.1](https://www.ncbi.nlm.nih.gov/entrez/viewer.fcgi?db=nucleotide&id=2062814713) | CCCCAAGATTCTCTCAGCCC  ACCCAAGTTAGAAAGCGCCA | 163 | 60.0 |
| CCR1 | [XM_004018514.5](https://www.ncbi.nlm.nih.gov/entrez/viewer.fcgi?db=nucleotide&id=2062814736) | CATCTACCTCCTCAACCTGGC  ACCAAAGGTGACAGTCCGAG | 241 | 60.0 |
| CXCL8 | [NM_001009401.2](https://www.ncbi.nlm.nih.gov/entrez/viewer.fcgi?db=nucleotide&id=427197617) | GACCCCAAGGAAAAGTGGGT  AGGAACTCGTGAATCCTGGC | 421 | 60.0 |
| CXCL11 | [XM_027971095.2](https://www.ncbi.nlm.nih.gov/entrez/viewer.fcgi?db=nucleotide&id=2062908420) | GCATGGCTATAGTCCTGGCT  GCCTATGCAAAGACACCGAC | 89 | 60.0 |
| CXCL12 | [XM_004021573.4](https://www.ncbi.nlm.nih.gov/entrez/viewer.fcgi?db=nucleotide&id=2062865107) | AAACTGTGCCCTCCAGATCG  CCACTTCAGCTTCGGGTCAA | 76 | 60.0 |
| CXCL13 | [XM_004009931.4](https://www.ncbi.nlm.nih.gov/entrez/viewer.fcgi?db=nucleotide&id=2062908533) | GCGTGGATGTCCAAACACAG  TGGCATCAGGCACTCCTTTT | 165 | 60.0 |
| CCL21 | [XM_004004119.4](https://www.ncbi.nlm.nih.gov/entrez/viewer.fcgi?db=nucleotide&id=2062821463) | TATCCTGTTCTCGCCTCGGA  CTGGGCTATGGCCCTTTTGA | 219 | 60.0 |
| CCL26 | [XM_004020976.5](https://www.ncbi.nlm.nih.gov/entrez/viewer.fcgi?db=nucleotide&id=2062858860) | GATGTGGCCAAGTTCTGCTG  CTTCGGCTGGGCACATACTT | 150 | 60.0 |
| CCL3 | [XM_004012448.5](https://www.ncbi.nlm.nih.gov/entrez/viewer.fcgi?db=nucleotide&id=2062756700) | CTCGCAAAATCGTAGCCGAC  CGGCCTCTTTTGGTCTGGAA | 82 | 60.0 |
| CCL3L | [XM_004013194.5](https://www.ncbi.nlm.nih.gov/entrez/viewer.fcgi?db=nucleotide&id=2062756703) | TTCGTGGACGACTATTACGAGA  CACGACGTCACACACTTCCA | 341 | 60.0 |
| CXCL6 | [XM_027971406.2](https://www.ncbi.nlm.nih.gov/entrez/viewer.fcgi?db=nucleotide&id=2062910304) | CCAAGGTGGAAGTGGTAGCC  TTCTTCCAATGCACGGTCCT | 141 | 60.0 |
| CX3CR1 | [XM_004018235.5](https://www.ncbi.nlm.nih.gov/entrez/viewer.fcgi?db=nucleotide&id=2062816855) | AGGCGGGAAAGTGTTCTGAG  GACATTGGCGGTAAGACCCA | 268 | 60.0 |
| TNFSF8 | [XM_004003977.5](https://www.ncbi.nlm.nih.gov/entrez/viewer.fcgi?db=nucleotide&id=2062819860) | CCGTCTCAAGACACAGCCAT  CGGGTTGGGAATAGAGTCCG | 174 | 60.0 |
| TNFRSF8 | [XM_027961470.2](https://www.ncbi.nlm.nih.gov/entrez/viewer.fcgi?db=nucleotide&id=2062862961) | GAGGAACACCATGGCTCACT  ATGCACTTGTCTAGGTCCGC | 159 | 60.0 |
| LIFR | [XM_042233841.1](https://www.ncbi.nlm.nih.gov/entrez/viewer.fcgi?db=nucleotide&id=2062798723) | CATGTACCAGCCTCAGGCAA  ACTGTTGCTGTCTGTGGACC | 220 | 60.0 |
