# Supplementary material for: Differential gene expression and immune cell infiltration in maedi-visna virus-infected lung tissues
Source: BMC Genomics. 2024 May 30;25:534. doi: 10.1186/s12864-024-10448-2 (PMC11141007; doi:10.1186/s12864-024-10448-2)
Supplement: Supplementary file 4 — Supplementary Material 4 [file 12864_2024_10448_MOESM4_ESM.docx]

Supplementary Table S2 Overview of sequencing.

| Sample | RawDatas | CleanData | Q20(%) | Q30(%) | N(%) | GC(%) |
| --- | --- | --- | --- | --- | --- | --- |
| CK-1 | 64831868 | 64585922 | 97.07 | 92.11 | 0 | 51.36 |
| CK-2 | 61762646 | 61544058 | 96.97 | 91.87 | 0 | 51.21 |
| CK-3 | 67278774 | 67064858 | 97.16 | 92.26 | 0 | 51.39 |
| VM-1-1 | 57843080 | 57681020 | 98.02 | 94.19 | 0 | 50.66 |
| VM-4-1 | 53804482 | 53610484 | 97.08 | 92.04 | 0 | 52.08 |
| VM-1_4 | 61167618 | 60979824 | 97.25 | 92.39 | 0 | 52.15 |
